# Inferring Depression and Its Semantic Underpinnings from Simple Lexical Choices

**DOI:** 10.1155/2024/3010831

**Published:** 2024-03-28

**Authors:** Line Kruse, Roberta Rocca, Mikkel Wallentin

**Affiliations:** ^1^Department of Linguistics, Cognitive Science and Semiotics, Aarhus University, Aarhus, Denmark; ^2^Interacting Minds Center (IMC), Aarhus University, Aarhus, Denmark; ^3^Center of Functionally Integrative Neuroscience (CFIN), Aarhus University Hospital, Aarhus, Denmark

## Abstract

Spatial demonstratives are highly frequent linguistic universals, with at least two contrastive expressions (proximal (“this”) vs. distal (“that”)) indicating physical, social, or functional proximity of the speaker to the referent object. Recent evidence based on the Demonstrative Choice Task (DCT), in which participants couple words with a spatial demonstrative with no context provided, suggests that demonstrative use is also indicative of experienced or emotional proximity to the self in an imagined mental space. As depression is characterized by increased and maladaptive focus on the self, the DCT may be a simple and reliable way to elicit behaviors that enable inference on the presence of severe depressive states and allow descriptions of the semantic characteristics of individual differences in such states. In two independent cross-sectional studies, including 775 and 879 participants, respectively, we showed that DCT-based classification models reliably capture semantic characteristics of experiential states that are predictive of self-reported depression symptom severity, as measured by PHQ-9. In both samples, DCT classifiers outperformed baseline models and replicated semantic patterns of negative affect previously observed to be associated with depression. This indicates that the paradigm captures semantic characteristics of the experiential states underlying depression symptoms and may be used to map individuals along a broad semantic space, potentially providing novel insights into individual differences in depressive states.

## 1. Introduction

Depression is characterized as a disorder of “self,” involving maladaptive distortions in the experiential and narrative self [1]. These alterations are mainly characterized by increased self-focused attention and highly negative self-representation [2]. While such alterations may manifest as similar symptoms across subjects (e.g., sleep disturbances flattened affect), there appears to be a gap between the observed or reported symptom profiles and the underlying experiential profiles of individuals with depressive disorder. A large heterogeneity in symptom profiles can be observed [3, 4], and even within individuals with similar symptom profiles, there is substantial variation in disorder trajectories [5], treatment efficiency [6], and comorbidity with other psycho- and somatic pathologies [7]. While identifying the presence of standard symptoms is crucial for diagnosis, there may be important differences in the experiential states underlying these symptoms that are not captured in standard symptom scales. Means to investigate and identify characteristics of the experiential state of individuals may contribute importantly to symptom profiling approaches and provide information about the relationship between symptoms and the underlying mental experiences and potentially individual differences hereof.

The present work was aimed at investigating the extent to which differences in mental states related to depression symptom severity can be captured with a simple language task and characterized in semantic terms. It is well acknowledged that language use and language processing are highly reflective of individual differences in personality traits [8–12], gender [11], mood [13, 14], stance detection [15–17], and demographic characteristics [18–21]). Differences in language use and processing have further proved to be effective markers of psychiatric symptoms, particularly in the case of depression [22–25], where even the most conservative classification models perform comparably to the standard of validated self-report scales of symptom severity [24] and clinical assessment [22]. This suggests that language features capture a substantial portion of depression symptom variance. In these models, increased use of negatively valenced word categories and first-person singular pronouns is consistently among the strongest differentiating features for depression classification [1, 24, 26–30], indicating increased and maladaptive self-focus. These findings reflect those observed in neuroimaging studies, where observed hyperactivation in emotional processing circuits for negative stimuli and hypoactivation for positive stimuli [31, 32] are enhanced when involving self-referential emotional processing [31, 33].

Recent evidence based on the Demonstrative Choice Task (DCT) indicates that the coupling of proximal (“this”) and distal (“that”) spatial demonstrative forms with nouns is indicative of individuals' experienced/emotional proximity to the target word. Spatial demonstratives are among the few language universals [34], and most languages have at least two forms, a proximal and distal form, delineating both a physical, functional, and social distinction between peripersonal and extrapersonal space [35]. The usage of spatial demonstratives is thus indicative of the position of the speaker relative to the referent in both a physical and experiential (psychological) space in any given context [36, 37]). The DCT [38] involves a binary forced choice between the proximal and distal demonstrative form for sequentially presented nouns. Each noun is presented in isolation, leaving no contextual anchors. In a large-scale DCT study, Rocca and Wallentin [38] showed that choices of proximal/distal demonstratives were highly structured across participants. Results indicated that demonstrative choices were structured according to the semantic characteristics of the items, where, for instance, words scoring high on features as *fearful*, *harm*, *unpleasant*, and *angry* were associated with more distal demonstrative responses, while nouns scoring high on *needs*, *pleasant*, *happy*, and *self* elicited higher proportions of proximal demonstrative forms. These findings hold across English and Spanish [39] and Danish and Italian [37] and suggest that choice of demonstrative forms not only reflects contextually bound clues about proximity in physical space but also carries information about the position of individuals in a nonphysical semantic space.

Capturing important dimensions concerning self-focused mental representations, the DCT may encode information relevant to inferring the presence of severe depression and may be a simple and effective tool to identify and study the structure of semantic representations underlying depression and other disorders of the self. The present study investigated whether maladaptive mental states related to depression can be reliably captured and described using the DCT. We hypothesized that (a) predictive models can reliably identify individuals with high depression symptom scores based on representations of their behavior in the DCT and (b) words that are *most* predictive in this task will map onto semantic dimensions that are traditionally associated with depression-related alterations of the experiential self (e.g., negative valence). The significance of these results would be twofold. First, the DCT may provide additional assessment tools for depression whose added value lies in *not* directly priming towards reflection on depressive symptoms, potentially reducing biases in self-report. Second, the DCT may make it possible to characterize individual differences in disorder states *within* clinical groups, providing insights into individuals' specific experiential profiles in ways that are not captured by standard symptom scales.

The study was conducted in two independent samples to assess robustness and replicability of the results. The replication procedure was preregistered prior to conducting study 2 (https://osf.io/bqhyg/).

## 2. Materials and Methods

The submitted study adheres to the procedure described in the preregistered protocol, adding only a few elements for further data scrutiny (see details in Supplementary Materials). The project was approved by the Institutional Review Board at Aarhus University.

### 2.1. Participants

The experiments were conducted on the online platform Prolific (https://www.prolific.co). All participants were native English speakers (age ≥ 18 years). No other inclusion criteria were defined. Subjects were excluded if they fulfilled at least one of three criteria indicating low effort: (1) reaction time (RT) below 300 ms. in more than 10% of the trials, (2) response (button) entropy below 0.80 indicating a consistent response pattern irrespective of the stimuli (see entropy equation in Supplementary Materials), and (3) more than 3 of 15 failed attention checks.

Study 1 included 1004 participants, of which 201 subjects were excluded due to missing data in either task or questionnaire responses. Additionally, 28 subjects were excluded based on the three low effort criteria, yielding a final sample of 775 participants (gender: 352 female, 412 male, 10 nonbinary, and 1 other; age: 159 were 18-29 years, 211 were 30-39 years, 147 were 40-49 years, 149 were 50-59 years, 107 were 60+ years, and 2 did not report age).

Study 2 included 1064 participants, of which 155 subjects were excluded due to missing data. Additionally, 30 participants were excluded based on the low effort criteria, yielding a final sample of 879 participants (gender: 410 female, 461 male, 6 nonbinary, and 2 other; age: 213 were 18-29 years, 268 were 30-39 years, 190 were 40-49 years, 111 were 50-59 years, 92 were 60+ years, and 2 were unreported).

### 2.2. Materials

#### 2.2.1. Demonstrative Choice Task (DCT)

**Figure 1:**
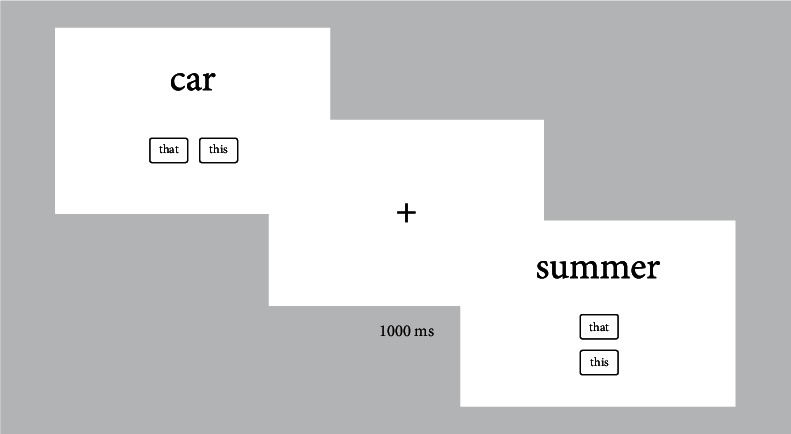
Demonstration of two trials in the DCT. In each trial, one noun is presented together with two response options. Response options change positions (4 different configurations) at random. Each stimulus is presented until the participant presses a button. Trials are separated by a fixation cross presented for 1000 ms.

Participants completed a 300-item Demonstrative Choice Task (DCT) adapted to the purpose from Rocca and Wallentin [38] (see Supplementary Experimental Procedures; full stimulus list in Figure S2). For each trial, an English noun was presented on the screen and participants were to match it with either a proximal (“this”) or distal (“that”) demonstrative forms by clicking one of two buttons presented below the stimulus (Figure 1). Trial order was randomized for each subject. Participants were unaware of the purpose of the study and informed that there was no incorrect answers and instructed to respond based on their immediate preference (Figure S1). For details on the experimental procedure, see Supplementary Materials.

#### 2.2.2. 9-Item Patient Health Questionnaire (PHQ-9)

Depression symptom severity was measured with the 9-item Patient Health Questionnaire (PHQ-9) [40]. PHQ-9 is a self-administered version of the PRIME-MD diagnostic instrument and measures each of the 9 DSM-IV criteria for depression on a 4-point Likert scale ranging from 0 (“not at all”) to 3 (“nearly every day”). The PHQ-9 instrument has demonstrated robust validity and reliability [41, 42], as well as sensitivity and specificity [43]. A sum score ≥ 10 (corresponding to moderate depression) was defined as threshold for classification of participants into the control or depression group.

### 2.3. Analysis

#### 2.3.1. Classification Models

Two logistic regression models were estimated, classifying the outcome group (control vs. depression) based on principal component (PC) representations of the DCT responses. The first model (mDCT) included only DCT responses. The second model (mDCT+Demo) included, in addition to DCT behavior, *gender* and *age* as predictors. These demographic features have shown to be associated with language usage [11, 18] as well as correlated with depression prevalence [44], and the second model addressed whether accounting for these variables could improve model performance. We additionally investigated whether performance of the DCT classifier improved, if restricting the training sample to subjects exhibiting test-retest reliability above 70% (see Supplementary Materials, Table S1 and Table S2).

Performance of the DCT classifiers was compared to two baseline models: one including only *gender* and *age* as predictors of the outcome group (mGenderAge) and one random baseline trained to classify a randomly shuffled version of the outcome group from DCT responses (mRandomBaseline). All models were trained on 70% of the data and evaluated on 30% of the data, stratified by the outcome group. Model performance was evaluated on out-of-sample classification accuracy, balanced between sensitivity (true positive rate) and specificity (true negative rate) and ROC AUC scores. Accuracy rate along with 95% confidence intervals for this rate was computed with a binomial test. *p* values for classification performance were computed with a one-sided test, evaluating whether performance was better than the no information rate, taken to be the largest class percentage in the data.

Data sensitivity analysis was performed for each model with nonparametric bootstrapping, evaluating robustness of model performance and feature importance to random data variation (see Supplementary Materials for details).

## 3. Results: Study 1

### 3.1. Descriptive Results

The median PHQ-9 sum score was 6 (mean = 7.6, min = 0, max = 25). 543 participants were categorized as control cases (PHQ‐9 < 10), and 243 were categorized as depression cases (PHQ‐9 ≥ 10) (Figure S3 in Supplementary Materials). The overall proportion of demonstrative choices across all participants (*n* responses = 232,500) was 48% for proximal and 52% for distal demonstrative forms. There was no difference in proportion of demonstrative choices between the control and depression groups (Figure S4).

### 3.2. Classification Performance

The two DCT models performed significantly better than chance level on classification of the depression group (Table 1). The DCT model (mDCT) exhibited an accuracy of .65, 95%CI = [.56, .72], *p* < .001, and ROC‐AUC = .67 (Figure S5). Adding demographic features to the DCT model (mDCT+Demo) improved performance slightly demonstrating an accuracy of .66, 95%CI = [.57, .74], *p* < .001, and ROC‐AUC = .67 (Figure S5).

Neither the random baseline model nor the demographic baseline model performed better than chance on classification of the depression group (Table 1 and Figure S5). ROC-AUC curves and confusion matrices are reported in Supplementary Materials (Figure S5 and Figure S6).

**Figure 2:**
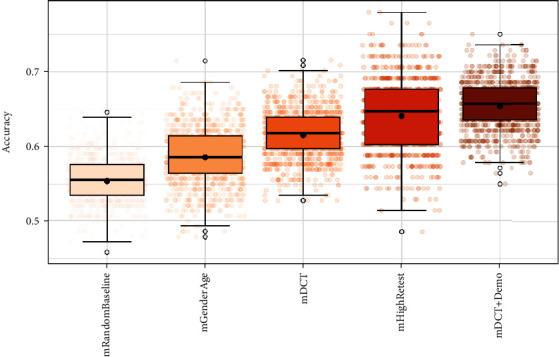
Study 1: accuracy estimates across *k* = 1000 bootstrapped model estimations. Black solid horizontal line indicates median accuracy. Black points indicate mean accuracy. Error bars indicate 1st and 3rd quartiles. Colored points indicate individual accuracy estimates for each bootstrapped sample. Mean accuracy estimates: mDCT + Demo = 65% (SD = 0.03), mHighRetest = 64% (SD = 0.04), mDCT = 62% (SD = 0.03), mGenderAge = 59% (SD = 0.04), and mRandomBaseline = 55% (SD = 0.03).

Bootstrapped data sensitivity analysis indicated that these patterns are robust to random variation in the data (Figure 2).

#### 3.2.1. Semantic Effects

**Figure 3:**
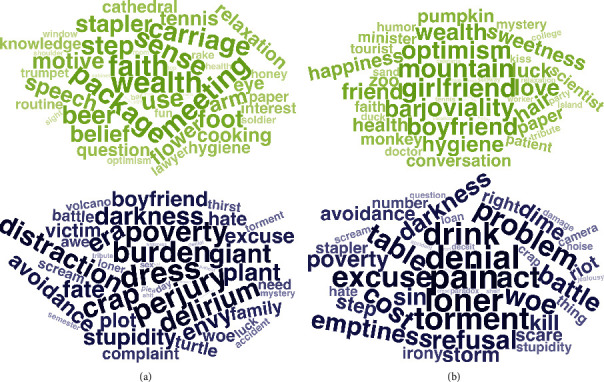
Study 1: the 50 DCT items with strongest positive (green) and negative (blue) regression coefficients for (a) mDCT and (b) mDCT+Demo. Positive effects indicate that choosing the proximal demonstrative for the given item increases the likelihood of being classified as control case. Negative regression effects indicate that choosing the proximal demonstrative for the given item increases the likelihood of being classified as depression case.

The fifty strongest positive and negative predictive DCT items for each model are visualized in Figure 3. A negative regression effect indicates that participants in the depression group were more likely than individuals in the control group to respond with a proximal demonstrative for the given item, while a positive regression effect indicates that they were more likely to respond with a distal demonstrative compared to the control group. Post hoc semantic analysis of the word effects in the best model (mDCT+Demo) showed a positive relationship between DCT item scores on semantic features of *trust*, *valence*, *dominance*, and *joy* and classification weights in the model. Contrary, results showed a negative relationship between DCT item classification weights and scores on the features *disgust*, *anger*, *sadness*, *arousal*, and *fear* (Figure S7, Table S3, Figure S8, and Table S4 in Supplementary Materials). These results indicate that participants in the depression group tended to respond with a distal demonstrative more often for highly negatively valenced words, while the opposite is true for highly positively valenced words.

## 4. Results: Study 2

### 4.1. Descriptive Results

The median PHQ-9 sum score was 6 (mean = 7.7, min = 0, max = 26). 588 participants were classified as control cases (PHQ‐9 < 10), and 291 were classified as depression cases (PHQ‐9 ≥ 10) (Figure S3). The overall proportion of demonstrative choices across all participants (*n* responses = 263,700) was 47% for proximal demonstrative and 53% for distal demonstrative. The proportion of demonstrative choices did not differ between the control and depression groups (Figure S4).

### 4.2. Classification Performance

The two DCT-based models performed significantly better than chance on classification of the depression group (Table 2). The DCT model (mDCT) exhibited a classification accuracy of .60, 97%CI = [.53, .68], *p* < .01, and ROC‐AUC = .58 (Figure S9). Including demographic features in the model slightly improved predictive performance; the mDCT+Demo model showed an accuracy of. 62, 95%CI = [.55, .70], *p* < .001, and ROC‐AUC = .62 (Figure S9).

Neither the random baseline model nor the demographic baseline models performed better than chance on classification of the depression group (Table 2 and Figure S9). ROC-AUC curves and confusion matrices of all models are reported in Supplementary Materials (Figure S9 and Figure S10).

**Figure 4:**
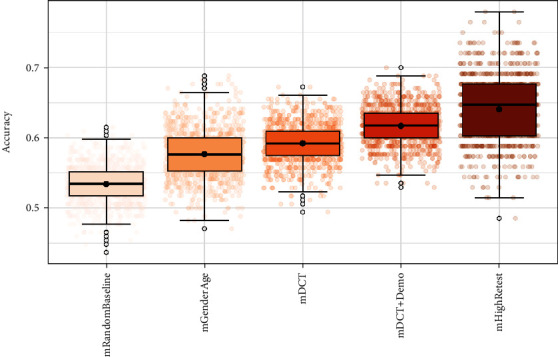
Study 2: accuracy estimates across *k* = 1000 bootstrapped model estimations. Black solid horizontal line indicates median accuracy. Black points indicate mean accuracy. Error bars indicate 1st and 3rd quartiles. Colored points indicate individual accuracy estimates for each bootstrapped training sample. Mean accuracy estimates: mHighRetest = 64% (SD = 0.04), mDCT + Gender = 62% (0.03), mDCT = 59% (SD = 0.04), mGenderAge = 58% (SD = 0.04), and mRandomBaseline = 53% (SD = 0.03).

Bootstrapped data sensitivity analysis showed that the patterns observed are robust to random data-induced variance (Figure 4).

#### 4.2.1. Semantic Effects

**Figure 5:**
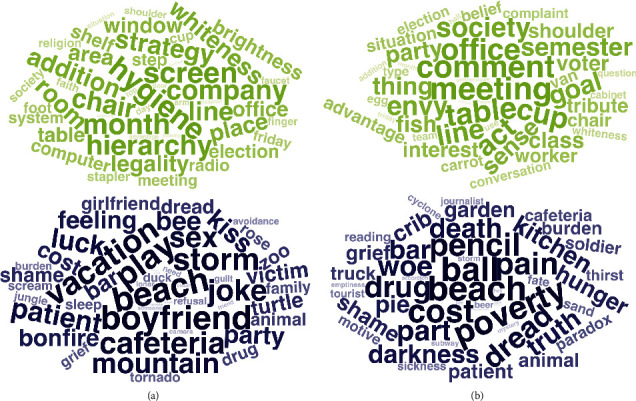
Study 2: the 50 DCT items with strongest positive (green) and negative (blue) regression coefficients for (a) mDCT and (b) mDCT+Demo. Positive effects indicate that choosing the proximal demonstrative for the given item increases the likelihood of being classified as control case. Negative regression effects indicate that choosing the proximal demonstrative for the given item increases the likelihood of being classified as depression case.

The fifty strongest positive and negative predictive DCT items for each model are visualized in Figure 5. Post hoc semantic analysis of the word effects in the best model (mDCT+Demo) showed a positive relationship between DCT item scores on the semantic features *valence* and *dominance* and item classification weights. Contrary, there was a negative relationship between item scores on the features *sadness*, *surprise*, *arousal*, and *fear* and item classification weights (Figure S7 and Figure S8 in Supplementary Materials).

#### 4.2.2. Correlation of Feature Importance in Study 1 and Study 2

The Pearson correlation of the bootstrapped word effects between study 1 and 2 was 0.35 (*p* < .001) for the mDCT model and 0.27 (*p* < .001) for the mDCT+Demo model (Figure S11 and Figure S12 in Supplementary Materials). In comparison, word effects of the mRandomBaseline model exhibited a correlation between study 1 and 2 of -0.08 (*p* = 1) (Figure S13).

## 5. Semantic Subject Profiles

Post hoc analyses were conducted to assess whether subject-wise semantic representations of DCT behavior were predictive of depression symptom severity. Each subject was ascribed a score on each of the 11 semantic features in the NRC-VAD lexicon [45, 46], calculated as the product of responses (-1 or 1) for each item and the item score on each semantic feature. Each participant was thus represented by a semantic vector of size 11, where low feature scores indicate larger proportion of proximal demonstrative choices for words scoring high on these dimensions, while high feature scores indicate larger proportions of distal demonstrative choices. A linear Bayesian model (BRM) was fitted for each semantic feature as predictor of the continuous PHQ-9 sum score, to evaluate the relationship between the semantic profile and depression symptom severity. Each BRM was estimated with 4 chains and 2000 iterations.

**Figure 6:**
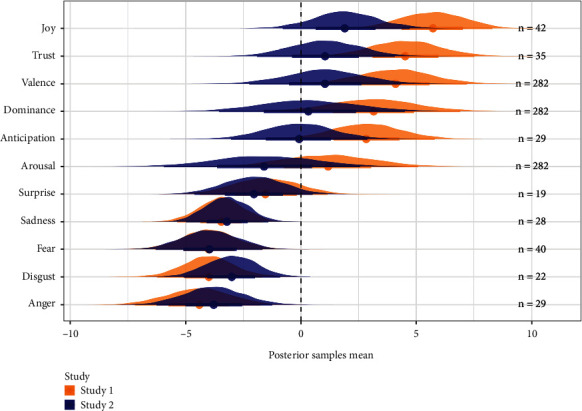
Posterior distributions of PHQ-9 sum scores predicted by subject-level semantic scores. A positive feature score indicates more distal responses for words scoring high on this feature. A negative feature score indicates more proximal responses for words scoring high on this feature. A positive regression effect indicates that higher feature scores predict higher PHQ-9 scores. A negative regression effect indicates that lower feature scores predict higher PHQ-9 scores. Orange: study 1. Blue: study 2. Black texts denote the number of words used in model estimation (the number of DCT items for which feature ratings were available).

For both study 1 and 2, results indicated a negative effect of *sadness*, *fear*, *disgust*, and *anger* on PHQ-9 sum score (Figure 6), indicating that more proximal demonstrative choices on words scoring high on these dimensions predicted higher PHQ-9 scores. Further, results showed a positive effect of *joy*, *trust*, and *valence* in study 1, indicating that more proximal demonstrative choices for words scoring high on these dimensions predicted lower PHQ-9 sum score. Posterior distributions for these positive effects in study 2 were in the same direction but overlapped with zero (Table S5 and Table S6).

## 6. Discussion

The present studies found that a simple lexical task, the DCT, elicits behaviors that can be used to infer self-reported depression status with classification accuracy ranging between 62% and 66% across two independent samples. Additionally, the DCT replicated semantic patterns of negative affect previously observed to be associated with depression [14, 24, 29, 47, 48]. Demonstrative choices for items scoring high on negative valence were consistently the strongest predictors in both studies, where proximal choices were predictive of the depression group and distal choices were predictive of the control group.

These results indicate that the DCT may be a useful tool to assist assessment of depression symptom severity, as it captures differences reliably related to depression in an indirect manner, i.e., without directly asking about depression. Such an approach may reduce potential biasing effects of meta-reflections in overt self-report symptom rating scales. While accuracy performance of the models were not as impressive as those observed for large social media based classification models, they are reliable across samples and recover semantic effects associated with depression. This indicates that it may be possible to adapt the model in ways that would optimize predictive performance, for instance, by assigning higher weights to particular word categories expected to have more predictive power, or replacing items found to be noninformative.

The semantic effects observed in the present study demonstrate the ability of the DCT to capture differences in semantic representations that are associated with self-reported depression symptoms. While we investigated semantic effects of the 11 emotional NRC-VAD features, expanding the paradigm to include a broader range of semantic features (i.e., not restricted to valence) could provide novel insights into the experiential states of depression. Similarly, the potential to create semantic subject profiles may allow investigations into individual differences in depressive states. The present study identified semantic characteristics shared across individuals with high depression symptom severity; however, it is likely that some semantic dimensions capture general depressive states shared across all patients (e.g. *valence*), while other dimensions may be descriptive of states that differ significantly between patients. Semantic categories as *social*, *body*, *money*, and *responsibility*, for instance, have exhibited strong relationships with individual differences in personality traits [11, 12] and may capture important aspects of individual differences in depressive experience. By mapping individuals along a broad set of semantic dimensions based on DCT behavior and computing individual deviations along each feature with respect to the group norm, the paradigm may improve our understanding of both homogeneous and heterogeneous characteristics of the experiential profiles underlying clinical symptoms. Further, such an approach may be extended to individual differences in other maladaptive states such as those associated with psychosis or personality disorders.

While the results show that structures in DCT behavior reliably relate to self-reported depression symptom severity, there are substantial amounts of unmodeled variation in behavior across individuals. Some variation is to be expected, as the task is binary and involves responses based on intuition rather than explicit reflective decisions. Additionally, the paradigm is sensitive to task context and transient mental states (e.g., participants may be more likely to respond with a proximal demonstrative for the item “Friday” if the task is conducted on a Friday). Thus, a series of trials is necessary for stable patterns to emerge. Importantly, some of the transient effects in DCT behavior may reflect important within-subject dynamics that are psychologically relevant (e.g., frequent mood changes or recurring anxiety states). Such sources of variation could potentially be dissociated from random noise in longitudinal DCT studies and would likely improve inference at the individual level. Additionally, obtaining more comprehensive semantic feature space along which DCT items can be scored would allow model inference based on semantic dimensions rather than individual items, reducing the impact of item-specific random variation on model performance.

The models presented in this work are based on self-reported depression symptoms, as indicated by the PHQ-9. While the PHQ-9 is a commonly used instrument to assess depression symptom severity, it is not a diagnostic tool and does not allow conclusions on the presence of clinical depression. Future work should aim to validate these models in a sample of clinically diagnosed patients. It is a general challenge that classification models can never be better than the objective against which they are evaluated. Subjective verbal reports and assessments of symptom severity are fundamental to clinical depression assessments and diagnoses, which are the gold standard of evaluation of any other model. Thus, DCT-based classification models cannot perform better than standard scales in identifying depression. What the present results suggest is that the paradigm may be a useful complementary tool to standard diagnostic procedures, as it captures information related to the presence of depression symptoms, and allow semantic analyses that could provide a more nuanced picture of individual disorder states.

## 7. Conclusions

The present results demonstrated that a simple lexical choice task reliably captures semantic characteristics of experiential states that are predictive of depression symptom severity across two independent samples. Future work may allow the mapping of individual differences in disorder states along a diverse set of semantic features and provide new insights into the specific experiential profiles underlying clinical symptoms and potential individual differences hereof.

## Figures and Tables

**Table 1:** Study 1: classification performance metrics.

Model	*n* PCs	Accuracy		95% CI		*p*		ROC AUC		Sensitivity		Specificity	
		Train	Test	Train	Test	Train	Test	Train	Test	Train	Test	Train	Test
mDCT	18	.61	.65	[.56, .67]	[.56, .72]	<.001	<.001	.67	.67	.61	.67	.62	.63
mDCT+Demo	**10**	**.62**	**.66**	**[.57, .67]**	**[.57, .74]**	**<.001**	**<.001**	**.66**	**.67**	**.61**	**.69**	**.63**	**.63**
mGenderAge	—	.58	.56	[.52, .63]	[.48, .65]	<.05	.07	.61	.59	.53	.54	.61	.59
mRandomBaseline	35	.65	.54	[.60, .70]	[.46, .62]	<.001	.18	.70	.49	.63	.56	.68	.53

The best performing model is highlighted in bold.

**Table 2:** Study 2: classification performance metrics.

Model	*n* PCs	Accuracy		95% CI		*p*		ROC AUC		Sensitivity		Specificity	
		Train	Test	Train	Test	Train	Test	Train	Test	Train	Test	Train	Test
mDCT	10	.59	.60	[.54, .64]	[.53, .68]	<.001	<.01	.58	.58	.57	.55	.61	.66
mDCT+Demo	**24**	**.63**	**.62**	**[.58, .68]**	**[.55, .70]**	**<.001**	**<.001**	**.70**	**.62**	**.66**	**.60**	**.60**	**.65**
mGenderAge	—	.59	.53	[.54, .64]	[.45, .61]	<.001	.25	.61	.60	.61	.58	.58	.48
mRandomBaseline	8	.55	.55	[.50, .60]	[.47, .63]	<.05	.10	.59	.52	.57	.59	.54	.51

The best performing model is highlighted in bold.

## Data Availability

The preregistered protocol, Supplementary Materials, and data along with scripts for analyses are available at the Open Science Framework (https://osf.io/bqhyg/).

## Supplementary Materials

Supplementary Materials Supplemental material includes 24 figures, 6 tables, and supplemental experimental procedures. Figure S1: participant instructions for the Demonstrative Choice Task. Figure S2: full stimuli list for the Demonstrative Choice Task. Table S1: study 1—classification performance metrics of all models. Table S2: study 2—classification performance metrics of all models. Figure S3: descriptive results: PHQ-9 sum score distribution by study. Figure S4: descriptive results: task response proportions by the study and outcome groups. Figure S5: study 1—ROC AUC scores for all models. Figure S6: study 1—confusion matrices for all models. Figure S7: post hoc Bayesian regression results—item classification coefficients predicted by semantic feature scores. Figure S8: post hoc Bayesian regression results—bootstrapped item classification coefficients predicted by semantic feature scores. Table S3: post hoc Bayesian regression results—item classification coefficients predicted by semantic feature scores (study 1). Table S4: post hoc Bayesian regression results—item classification coefficients predicted by semantic feature scores (study 2). Figure S9: study 2—ROC AUC scores for all models. Figure S10: study 2—confusion matrices for all models. Figure S11: correlation of bootstrapped word effects for model *mDCT* between study 1 and study 2. Figure S12: correlation of bootstrapped word effects for model *mDCT+Demo* between study 1 and study 2. Figure S13: correlation of bootstrapped word effects for model *mRandomBaseline* between study 1 and study 2. Table S5: post hoc Bayesian regression results—PHQ-9 sum scores predicted by semantic subject profiles (study 1). Table S6: post hoc Bayesian regression results—PHQ-9 sum scores predicted by semantic subject profiles (study 2). Supplementary Experimental Procedures: extended materials and methods section, including details on stimuli selection, PCA analysis, and post hoc sensitivity and semantic analyses.

## References

[1] Newell E. E., McCoy S. K., Newman M. L., Wellman J. D., Gardner S. K. (2018). You sound so down: capturing depressed affect through depressed language. *Journal of Language and Social Psychology*.

[2] Davey C. G., Harrison B. J. (2022). The self on its axis: a framework for understanding depression. *Translational Psychiatry*.

[3] Hannon K., Easley T., Zhang W. (2022). *Heterogeneity in depression: evidence for distinct clinical and neurobiological profiles*.

[4] Buch A. M., Liston C. (2021). Dissecting diagnostic heterogeneity in depression by integrating neuroimaging and genetics. *Neuropsychopharmacology*.

[5] Musliner K. L., Munk-Olsen T., Eaton W. W., Zandi P. P. (2016). Heterogeneity in long-term trajectories of depressive symptoms: patterns, predictors and outcomes. *Journal of Affective Disorders*.

[6] Carter G. C., Cantrell R. A., Zarotsky V. (2012). Comprehensive review of factors implicated in the heterogeneity of response in depression. *Depression and Anxiety*.

[7] Steffen A., Nübel J., Jacobi F., Bätzing J., Holstiege J. (2020). Mental and somatic comorbidity of depression: a comprehensive cross-sectional analysis of 202 diagnosis groups using German nationwide ambulatory claims data. *BMC Psychiatry*.

[8] Christian H., Suhartono D., Chowanda A., Zamli K. Z. (2021). Text based personality prediction from multiple social media data sources using pre-trained language model and model averaging. *Journal of Big Data*.

[9] Hassanein M., Hussein W., Rady S., Gharib T. F. Predicting personality traits from social media using text semantics.

[10] Kwantes P. J., Derbentseva N., Lam Q., Vartanian O., Marmurek H. H. C. (2016). Assessing the big five personality traits with latent semantic analysis. *Personality and Individual Differences*.

[11] Andrew Schwartz H., Eichstaedt J. C., Kern M. L. (2013). Personality, gender, and age in the language of social media: the open-vocabulary approach. *PLoS ONE*.

[12] Yarkoni T. (2010). Personality in 100,000 words: a large-scale analysis of personality and word use among bloggers. *Journal of Research in Personality*.

[13] Behdarvandirad S., Karami H. (2022). Depression, neuroticism, extraversion and pronoun use in first and foreign languages following mood induction. *Language Sciences*.

[14] Chen X., Sykora M. D., Jackson T. W., Elayan S. (2018). What about mood swings: identifying depression on twitter with temporal measures of emotions. *Companion Proceedings of the The Web Conference 2018. WWW’18*.

[15] Kawintiranon K., Singh L. (2021). Knowledge enhanced masked language model for stance detection. *Proceedings of the 2021 Conference of the North American Chapter of the Association for Computational Linguistics: Human Language Technologies*.

[16] Grimminger L., Klinger R. (2021). Hate towards the political opponent: a Twitter corpus study of the 2020 US elections on the basis of offensive speech and stance detection. https://arxiv.org/abs/2103.01664.

[17] Kawintiranon K., Singh L. (2022). PoliBERTweet: a pre-trained language model for analyzing political content on Twitter. *Proceedings of the Thirteenth Language Resources and Evaluation Conference*.

[18] Sap M., Park G., Eichstaedt J. Developing age and gender predictive lexica over social media.

[19] Tigunova A., Yates A., Mirza P., Weikum G. Listening between the lines: learning personal attributes from conversations.

[20] Wang Z., Hale S., Adelani D. I. (2019). Demographic inference and representative population estimates from multilingual social media data. *The World Wide Web Conference. WWW’19*.

[21] Lai Y., Yijun S., Xue C., Zha D., Jensen C. S., Lim E.-P., Yang D.-N. (2021). Neural demographic prediction in social media with deep multi-view multi-task learning. *Database Systems for Advanced Applications*.

[22] Guntuku S. C., Yaden D. B., Kern M. L., Ungar L. H., Eichstaedt J. C. (2017). Detecting depression and mental illness on social media: an integrative review. *Current Opinion in Behavioral Sciences. Big Data in the Behavioural Sciences*.

[23] Kour H., Gupta M. K. (2022). Predicting the language of depression from multivariate twitter data using a feature-rich hybrid deep learning model. *Concurrency and Computation: Practice and Experience*.

[24] Eichstaedt J. C., Smith R. J., Merchant R. M. (2018). Facebook language predicts depression in medical records. *Proceedings of the National Academy of Sciences*.

[25] Tsugawa S., Kikuchi Y., Kishino F., Nakajima K., Itoh Y., Ohsaki H. Recognizing depression from Twitter activity.

[26] Rude S., Gortner E.-M., Pennebaker J. (2004). Language use of depressed and depression-vulnerable college students. *Cognition and Emotion*.

[27] Trotzek M., Koitka S., Friedrich C. M. (2018). Utilizing neural networks and linguistic metadata for early detection of depression indications in text sequences. *IEEE Transactions on Knowledge and Data Engineering*.

[28] Edwards T.’m., Holtzman N. S. (2017). A meta-analysis of correlations between depression and first person singular pronoun use. *Journal of Research in Personality*.

[29] Preoţiuc-Pietro D., Eichstaedt J., Park G. (2015). The role of personality, age, and gender in tweeting about mental illness. *Proceedings of the 2nd Workshop on Computational Linguistics and Clinical Psychology: From Linguistic Signal to Clinical Reality. CLPsych 2015*.

[30] Begum S. R., Sait S. Y. Effective techniques for depression detection on social media: a comprehensive review.

[31] Klumpp H., Deldin P. (2010). Review of brain functioning in depression for semantic processing and verbal fluency. *International Journal of Psychophysiology. Psychophysiology of Language Processes in Psychopathology*.

[32] Groenewold N. A., Opmeer E. M., de Jonge P., Aleman A., Costafreda S. G. (2013). Emotional valence modulates brain functional abnormalities in depression: evidence from a meta-analysis of fMRI studies. *Neuroscience & Biobehavioral Reviews*.

[33] Miskowiak K. W., Larsen J. E., Harmer C. J. (2018). Is negative self-referent bias an endophenotype for depression? An fMRI study of emotional self-referent words in twins at high vs. low risk of depression. *Journal of Affective Disorders*.

[34] Diessel H. (2014). Demonstratives, frames of reference, and semantic universals of space. *Language and Linguistics Compass*.

[35] van Schuppen L., van Krieken K., Sanders J. (2019). Deictic navigation network: linguistic viewpoint disturbances in schizophrenia. *Frontiers in Psychology*.

[36] Coventry K. R., Griffiths D., Hamilton C. J. (2014). Spatial demonstratives and perceptual space: describing and remembering object location. *Cognitive Psychology*.

[37] Rocca R., Tylén K., Wallentin M. (2019). This shoe, that tiger: Semantic properties reflecting manual affordances of the referent modulate demonstrative use. *PloS One*.

[38] Rocca R., Wallentin M. (2020). Demonstrative reference and semantic space: a large-scale demonstrative choice task study. *Frontiers in Psychology*.

[39] Todisco E., Rocca R., Wallentin M. (2021). The semantics of spatial demonstratives in Spanish: a demonstrative choice task study. *Language and Cognition*.

[40] Kroenke K., Spitzer R. L., Williams J. B. W. (2001). The PHQ-9: validity of a brief depression severity measure. *Journal of General Internal Medicine*.

[41] Cameron I. M., Crawford J. R., Lawton K., Reid I. C. (2008). Psychometric comparison of PHQ-9 and HADS for measuring depression severity in primary care. *British Journal of General Practice*.

[42] Maroufizadeh S., Omani-Samani R., Almasi-Hashiani A., Amini P., Sepidarkish M. (2019). The reliability and validity of the Patient Health Questionnaire-9 (PHQ-9) and PHQ-2 in patients with infertility. *Reproductive Health*.

[43] Levis B., Benedetti A., Thombs B. D. (2019). Accuracy of Patient Health Questionnaire-9 (PHQ-9) for screening to detect major depression: individual participant data meta-analysis. *British Medical Journal*.

[44] National Institute of Mental Health (NIMH) Major Depression. https://www.nimh.nih.gov/health/statistics/major-depression.

[45] Mohammad S. M. (2022). *Word Affect Intensities*. https://arxiv.org/abs/1704.08798.

[46] Mohammad S. (2018). Obtaining reliable human ratings of valence, arousal, and dominance for 20,000 English words. *Proceedings of the 56th Annual Meeting of the Association for Computational Linguistics (Volume 1: Long Papers). ACL 2018*.

[47] Liu T., Meyerhoff J., Eichstaedt J. C. (2022). The relationship between text message sentiment and self-reported depression. *Journal of Affective Disorders*.

[48] De Choudhury M., Gamon M., Counts S., Horvitz E. (2013). Predicting depression via social media. *Proceedings of the International AAAI Conference on Web and Social Media*.

[49] Kruse L., Wallentin M., Rocca R. (2023). *Inferring depression and its semantic underpinnings from simple lexical choices*.

