# Supplementary material for: Inferring Depression and Its Semantic Underpinnings from Simple Lexical Choices
Source: Depress Anxiety. 2024 Mar 28;2024:3010831. doi: 10.1155/2024/3010831 (PMC11918855; doi:10.1155/2024/3010831)
Supplement: Supplementary Materials — Supplemental material includes 24 figures, 6 tables, and supplemental experimental procedures. Figure S1: participant instructions for the Demonstrative Choice Task. Figure S2: full stimuli list for the Demonstrative Choice Task. Table S1: study 1—classification performance metrics of all models. Table S2: study 2—classification performance metrics of all models. Figure S3: descriptive results: PHQ-9 sum score distribution by study. Figure S4: descriptive results: task response proportions by the study and outcome groups. Figure S5: study 1—ROC AUC scores for all models. Figure S6: study 1—confusion matrices for all models. Figure S7: post hoc Bayesian regression results—item classification coefficients predicted by semantic feature scores. Figure S8: post hoc Bayesian regression results—bootstrapped item classification coefficients predicted by semantic feature scores. Table S3: post hoc Bayesian regression results—item classification coefficients predicted by semantic feature scores (study 1). Table S4: post hoc Bayesian regression results—item classification coefficients predicted by semantic feature scores (study 2). Figure S9: study 2—ROC AUC scores for all models. Figure S10: study 2—confusion matrices for all models. Figure S11: correlation of bootstrapped word effects for model mDCT between study 1 and study 2. Figure S12: correlation of bootstrapped word effects for model mDCT+Demo between study 1 and study 2. Figure S13: correlation of bootstrapped word effects for model mRandomBaseline between study 1 and study 2. Table S5: post hoc Bayesian regression results—PHQ-9 sum scores predicted by semantic subject profiles (study 1). Table S6: post hoc Bayesian regression results—PHQ-9 sum scores predicted by semantic subject profiles (study 2). Supplementary Experimental Procedures: extended materials and methods section, including details on stimuli selection, PCA analysis, and post hoc sensitivity and semantic analyses. [file 3010831.f1.pdf]

Supplementary Materials
Inferring Depression and Its Semantic Underpinnings from Simple Lexical Choices

Line Kruse, Roberta Rocca, Mikkel Wallentin

Figure S1: DCT Task Instructions
Figure S2: DCT Stimuli List
Table S1: Study 1 - Classification Performance Metrics of All Models
Table S2: Study 2 - Classification Performance Metrics of All Models
Figure S3: PHQ-9 Sum Score Distribution by Study
Figure S4: Proportion Proximal vs. Distal Demonstrative Responses by Study and Group
Figure S5: Study 1 - ROC AUC Scores for All Models
Figure S6: Study 1 - Confusion Matrices for All Models
Figure S7: Item Classification Coefficients Predicted by Semantic Feature Scores
Figure S8: Bootstrapped Item Classification Coefficients Predicted by Semantic Feature Scores
Table S3: Study 1 - Item Classification Coefficients Predicted by Semantic Feature Scores
Table S4: Study 2 - Item Classification Coefficients Predicted by Semantic Feature Scores
Figure S9: Study 2 - ROC AUC Scores for All Models
Figure S10: Study 2 - Confusion Matrices for All Models
Figure S11: Correlation of Bootstrapped Item Classification Coefficients for *mDCT* in Study 1 and Study 2
Figure S12: Correlation of Bootstrapped Item Classification Coefficients for *mDCT+Demo* in Study 1 and Study 2
Figure S13: Correlation of Bootstrapped Item Classification Coefficients for *mRandomBaseline* in Study 1 and Study 2
Table S5: Study 1 - PHQ-9 Sum Scores Predicted by Semantic Subject Profiles
Table S6: Study 2 - PHQ-9 Sum Scores Predicted by Semantic Subject Profiles
Supplementary Experimental Procedures

Figure S1: DCT Task Instructions

Thank you for participating in our survey on lexical choice in demonstrative reference.

In what follows, you will be presented with a series of 300 words, and asked to match them with either "this" or "that". There is no specific rule to follow: just make your choice based on your first and immediate preference.

Notice that the position of "this" and "that" response buttons changes randomly.

Random attention checks will appear, in which you are asked to indicate the last word you saw.

The experiment consists of three blocks of 100 words. Between each block, you can take a break if you want to.

After the words, you will be asked 45 questions related to your personality type and mood during the past few weeks.

The survey will take around 20 minutes in total. A progress bar above each question will enable you to keep track of the unfolding of the survey.

Figure 1

Figure S2: DCT Stimuli List

love, boyfriend, home, friend, family, game, football, play, team, basketball, girlfriend, win, sport, tennis, band, fun, coolness, hell, hate, shit, crap, care, stupidity, movie, computer, drug, world, death, society, religion, human, faith, type, college, group, situation, class, problem, goodness, health, poverty, ease, conversation, avoidance, scare, pain, dress, awe, line, lake, radio, kill, goal, right, greatness, interest, addition, top, routine, package, whiteness, reading, darkness, money, drink, hunger, boy, question, freedom, thirst, change, refusal, need, day, Friday, clue, system, step, comment, rent, act, distraction, life, someone, part, thought, feeling, abyss, sex, sweetness, screen, study, meeting, high, sleep, relaxation, office, cooking, homesickness, thing, place, company, window, number, shoe, room, chair, area, month, eye, car, pencil, door, toothbrush, cup, paper, lamp, plant, flower, sin, excuse, guilt, jealousy, plea, shame, camera, choir, criminal, garden, grief, complaint, hygiene, accordion, delirium, emptiness, plot, riot, torment, damage, hierarchy, sense, snake, stampede, truck, use, bribe, denial, dog, dread, elm, fate, kiss, knowledge, humor, animal, deceit, loner, satire, scream, sickness, actor, battle, cyclone, perjury, victim, woe, coffee, cafeteria, hall, cab, storm, turtle, wonder, patient, tornado, trumpet, finger, hair, pineapple, businessman, happiness, joviality, minister, vacation, speech, stapler, worker, arm, egg, ham, joke, trust, summer, crib, engineer, ketchup, kitchen, shelf, testimony, table, tribute, ball, beach, couple, dime, dinner, journalist, monkey, mystery, optimism, party, store, truth, belief, cabinet, field, volcano, color, envy, firework, lawyer, paradox, bonfire, election, cost, harp, highway, pie, rake, rose, slight, tourist, cathedral, fish, honey, pilot, scientist, shoulder, subway, voter, zoo, analogy, chicken, court, foot, irony, mountain, squeal, stone, duck, island, jungle, legality, accident, artist, boat, era, motive, soldier, van, debate, diplomat, guard, brightness, army, hotel, lemonade, beer, law, eggplant, bee, carriage, burden, noise, semester, wealth, nose, loan, bar, advantage, strategy, hospital, carrot, luck, faucet, pumpkin, giant, cloud, doctor, sand

Figure 2: Full list of unique nouns (n=290) included in the DCT.

Table S1: Study 1 - Classification Performance Metrics of All Models

Model	n PCs	Accuracy		95% CI		p		ROC AUC		Sensitivity		Specificity	
		train	test	train	test	train	test	train	test	train	test	train	test
mDCT	18	.61	.65	[.56, .67]	[.56, .72]	<.001	<.001	.67	.67	.61	.67	.62	.63
**mDCT+Demo**	**10**	**.62**	**.66**	**[.57, .67]**	**[.57, .74]**	**<.001**	**<.001**	**.66**	**.67**	**.61**	**.69**	**.63**	**.63**
**mHighRetest**	**2**	**.64**	**.74**	**[.56, .71]**	**[.61, .84]**	**<.001**	**<.001**	**.65**	**.79**	**.67**	**.79**	**.60**	**.68**
mGenderAge	-	.58	.56	[.52, .63]	[.48, .65]	<.05	.07	.61	.59	.53	.54	.61	.59
mRandomBaseline	35	.65	.54	[.60, .70]	[.46, .62]	<.001	.18	.70	.49	.63	.56	.68	.53

Table 1: Study 1

Table S2: Study 2 - Classification Performance Metrics of All Models

Model	n PCs	Accuracy		95% CI		p		ROC AUC		Sensitivity		Specificity	
		train	test	train	test	train	test	train	test	train	test	train	test
mDCT	10	.59	.60	[.54, .64]	[.53, .68]	<.001	<.01	.58	.58	.57	.55	.61	.66
**mDCT+Demo**	**24**	**.63**	**.62**	**[.58, .68]**	**[.55, .70]**	**<.001**	**<.001**	**.70**	**.62**	**.66**	**.60**	**.60**	**.65**
mHighRetest		.75	.61	[.69, .81]	[.50, .71]	<.001	<.05	.82	.60	.75	.62	.76	.60
mGenderAge	-	.59	.53	[.54, .64]	[.45, .61]	<.001	.25	.61	.60	.61	.58	.58	.48
mRandomBaseline	8	.55	.55	[.50, .60]	[.47, .63]	<.05	.10	.59	.52	.57	.59	.54	.51

Table 2: Study 2

Figure S3:PHQ-9 Sum Score Distribution by Study

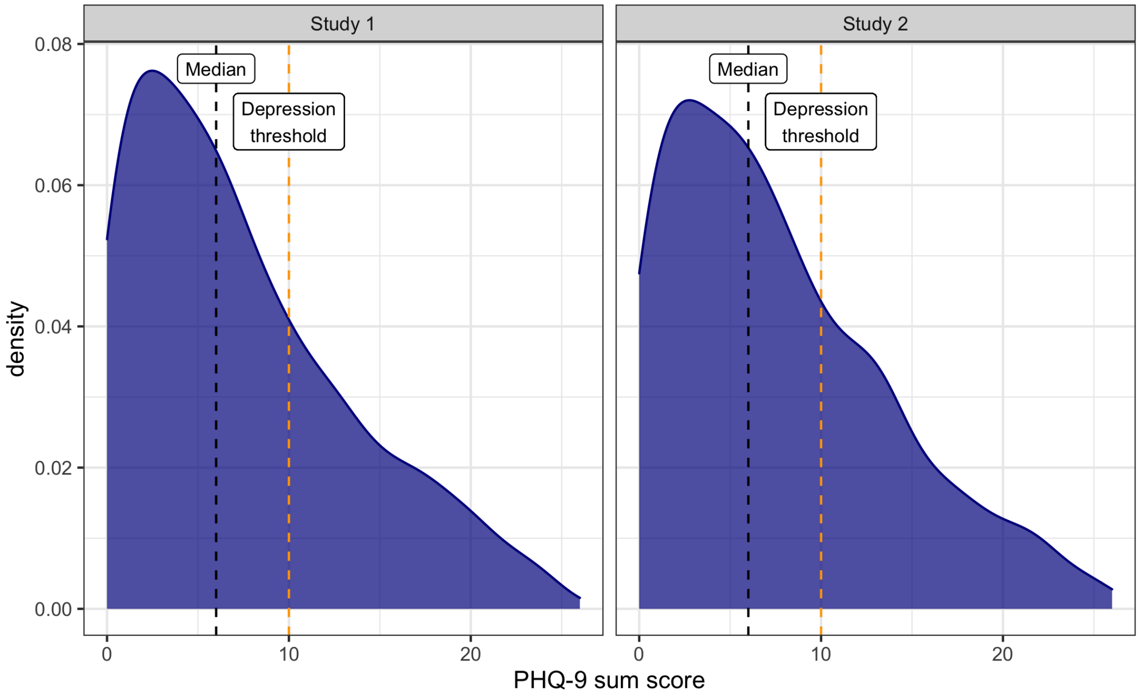

Figure 3: PHQ-9 sum score distribution for study 1 (left) and study 2 (right). Black dotted lines indicate the median sum scores. Orange dotted lines indicate the depression threshold score (10).

Figure S4: Proportion Proximal vs. Distal Demonstrative Responses by Study and Group

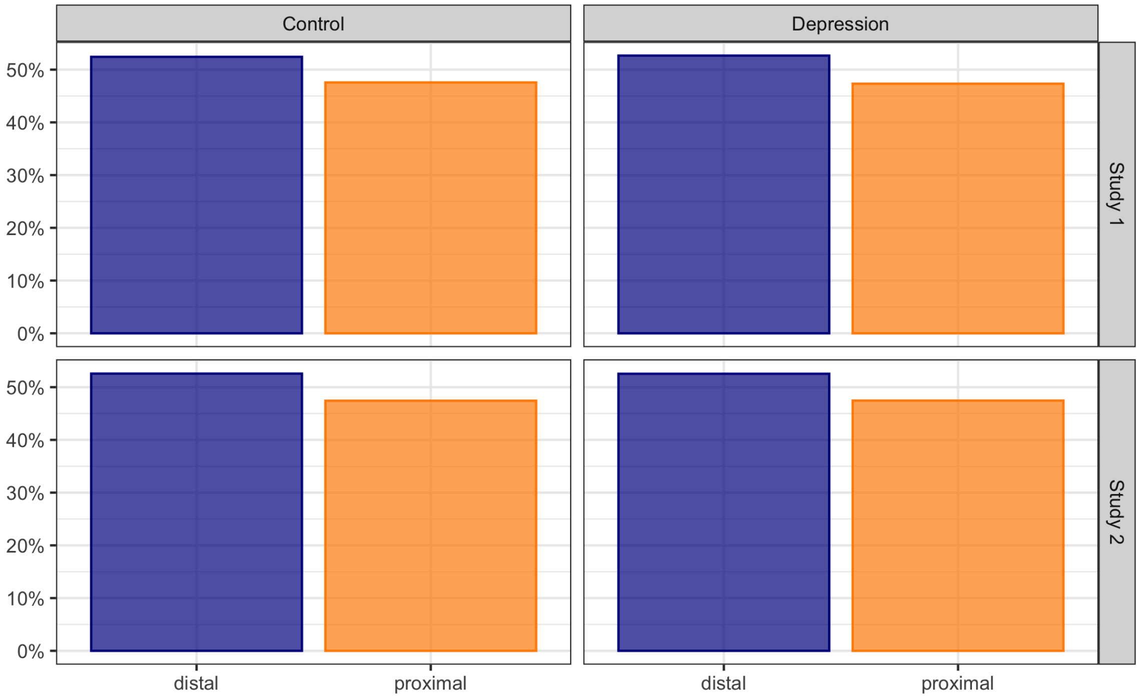

Figure 4: Percentage of proximal and distal demonstrative choices by group (left: control, right: depression) and study (top: study 1, bottom: study 2). Blue: distal demonstrative (”that”), orange: proximal demonstrative (”this”)

.

Figure S5: Study 1 - ROC AUC Scores for All Models

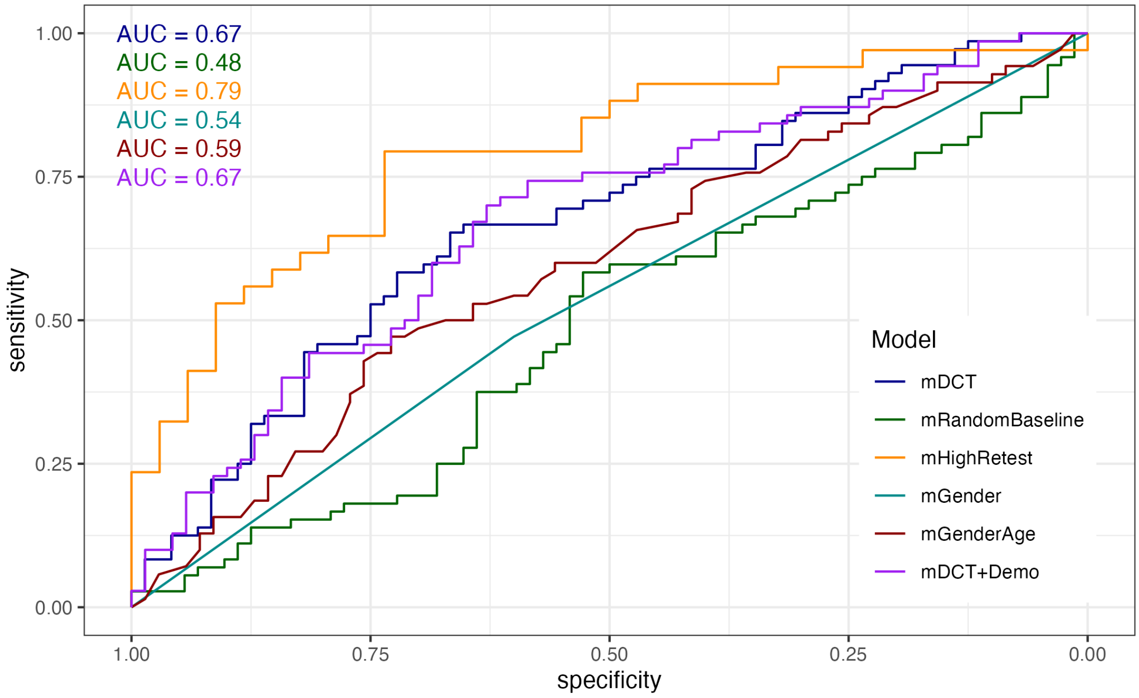

Figure 5: Study 1. Receiver Operated Curve (ROC) and Area Under the Curve (AUC) by model.

Figure S6: Study 1 - Confusion Matrices for All Models

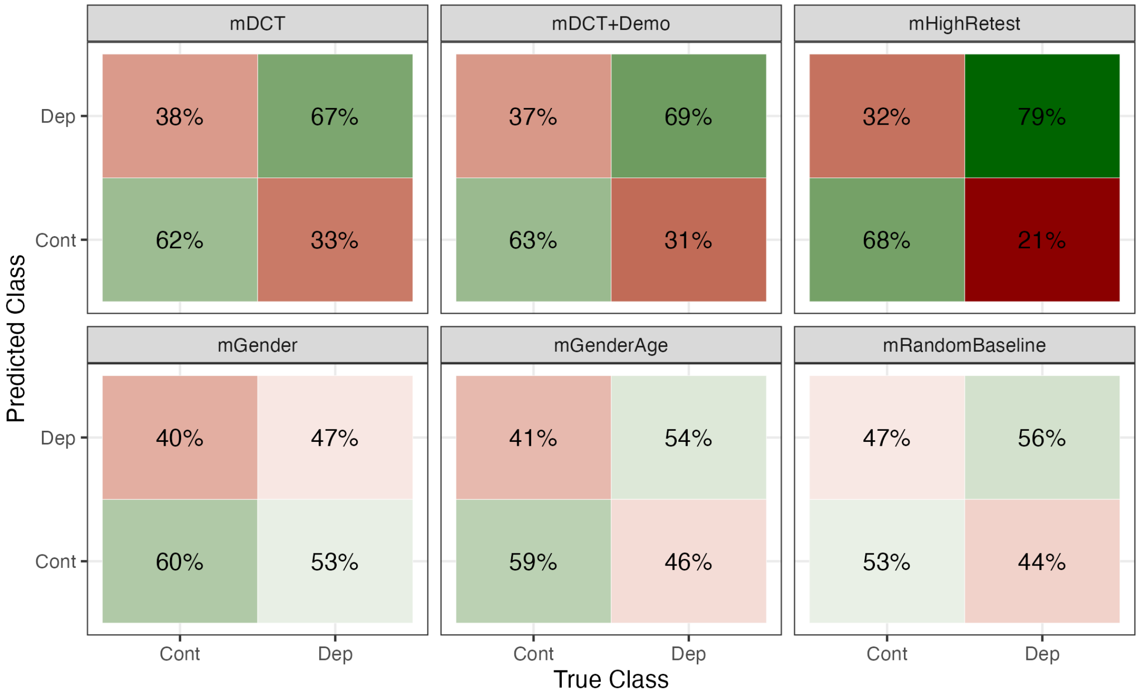

Figure 6: Study 1. Classification confusion matrices. Percentage correct predictions with respect to the true class. Red: lower values, green: higher values.

Figure S7: Item Classification Coefficients Predicted by Semantic Feature Scores

Results of the post-hoc semantic analysis of the word effects suggested that words scoring high on *trust*, *valence*, *dominance*, and *joy* were associated with higher classification coefficients, i.e., higher likelihood of distal demonstrative choices in depression group than control group, while words scoring high on *fear*, *arousal*, *surprise*, *sadness* and *anger* were associated with lower classification effects, i.e., higher likelihood of proximal demonstrative choices in depression group than control group (Figure S7, Table S**??**, and Table S**??**). Results indicate that the direction of effects replicate across study 1 and study 2 (except for *anticipation* and *disgust*), however, the effect of *fear*, *trust*, *joy*, and *anger* in study 2 appear to be less robust than for study 1. The same effects were observed for the averaged bootstrapped word effects in both study 1 and 2 (Figure S8) suggesting that these semantic effects are robust to random data-induced variability.

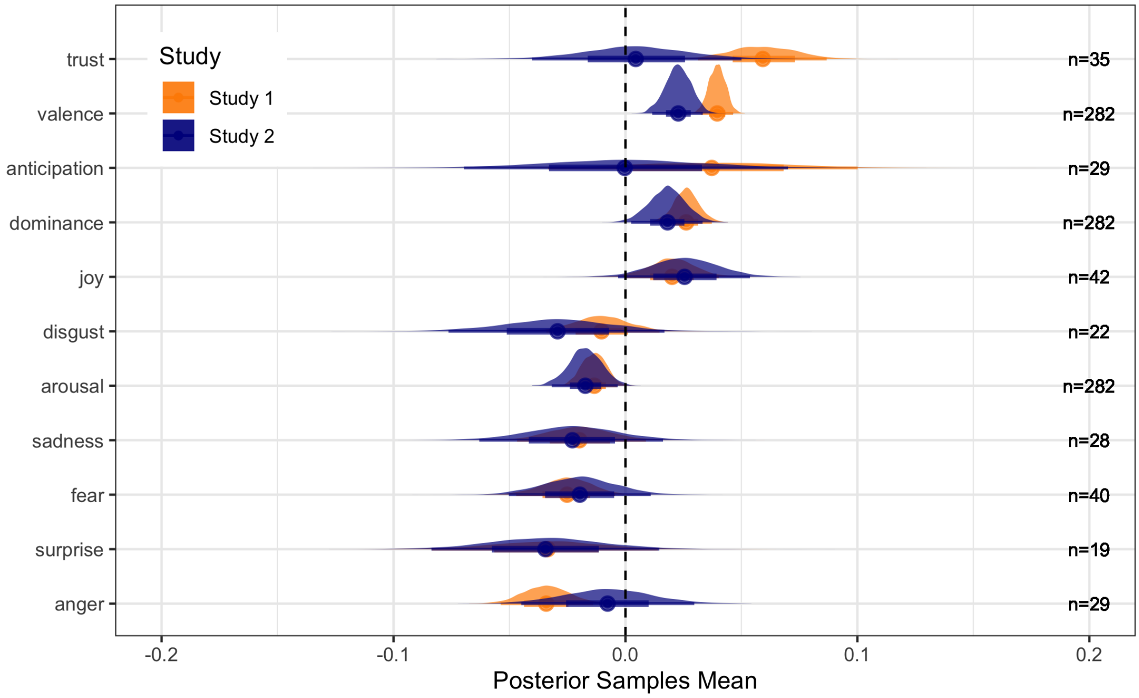

Figure 7: Posterior distributions of word-level classification coefficients (mDCT+Demo) predicted by semantic feature scores. Positive effects indicate that higher semantic feature scores predict higher classification effect of the word (positive classification effect). Negative effects indicate that higher semantic feature scores predict lower classification effect of the word (negative classification effect). Orange: study 1. Blue: study 2. Black text denote the number of words used in model estimation (the number of DCT items for which feature ratings were available).

Figure S8: Bootstrapped Item Classification Coefficients Predicted by Semantic Feature Scores

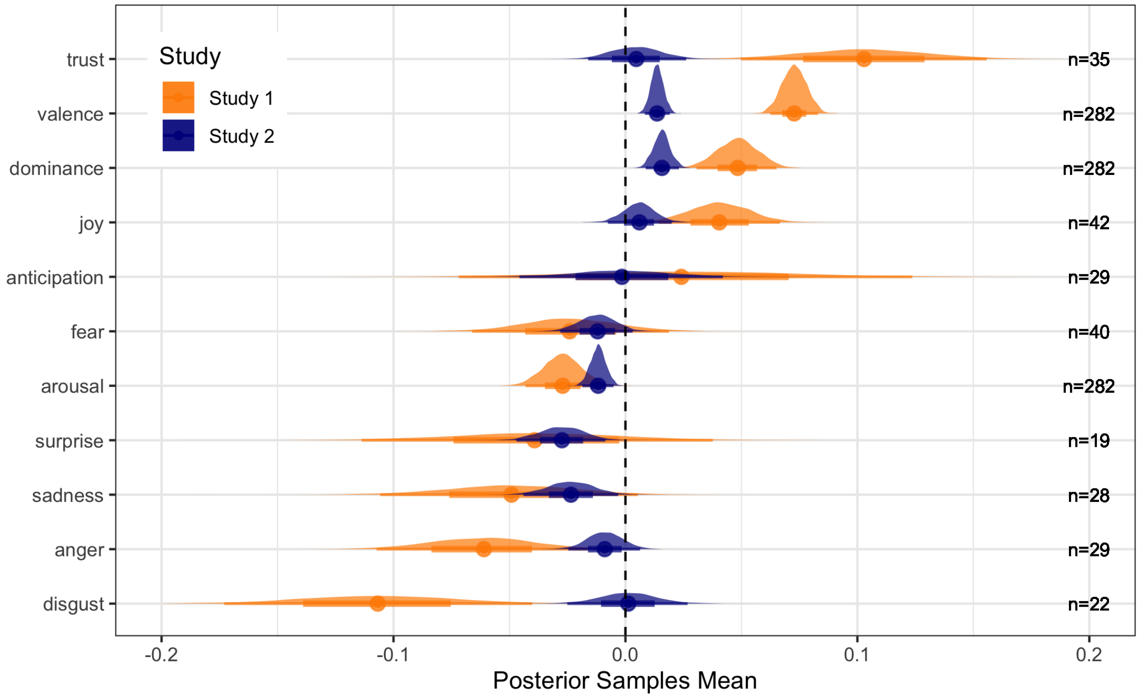

Figure 8: Posterior distributions of word-level classification effects (averaged across bootstrapped mDCT+Demo estimation) predicted by semantic feature scores. Positive effects indicate that higher semantic feature scores predict higher classification effect of the word (positive classification effect). Negative effects indicate that higher semantic feature scores predict lower classification effect of the word (negative classification effect). Orange: study 1. Blue: study 2. Black text denote the number of words used in model estimation (the number of DCT items for which feature ratings were available).

Table S3: Study 1 - Item Classification Coefficients Predicted by Semantic Feature Scores.

Feature	Estimate	Error	95% CI.l	95% CI.u
Valence	0.04	0.00	0.03	0.05
Arousal	-0.01	0.01	-0.02	-0.00
Dominance	0.03	0.01	0.02	0.04
Anger	-0.03	0.01	-0.05	-0.02
Anticipation	0.04	0.03	-0.03	0.10
Disgust	-0.01	0.01	-0.04	0.01
Fear	-0.03	0.01	-0.05	-0.00
Joy	0.02	0.01	0.00	0.04
Sadness	-0.02	0.01	-0.05	0.01
Surprise	-0.03	0.02	-0.08	0.01
Trust	0.06	0.01	0.03	0.09

Table 3: Study 1. Results of linear BRM predicting word-level classification coefficients from semantic feature scores. Each model was estimated separately.

Table S4: Study 2 - Item Classification Coefficients Predicted by Semantic Feature Scores.

Feature	Estimate	Error	95% CI.l	95% CI.u
Valence	0.02	0.01	0.01	0.03
Arousal	-0.02	0.01	-0.03	0.00
Dominance	0.02	0.01	0.00	0.03
Anger	-0.01	0.02	-0.04	0.03
Anticipation	-0.00	0.04	-0.07	0.07
Disgust	-0.03	0.02	-0.08	0.02
Fear	-0.02	0.02	-0.05	0.01
Joy	0.03	0.01	-0.00	0.05
Sadness	-0.02	0.02	-0.06	0.02
Surprise	-0.03	0.02	-0.08	0.02
Trust	0.00	0.02	-0.04	0.05

Table 4: Study 2. Results of linear BRM predicting word-level classification coefficients from semantic feature scores. Each model was estimated separately.

Figure S9: Study 2 - ROC AUC Scores for All Models

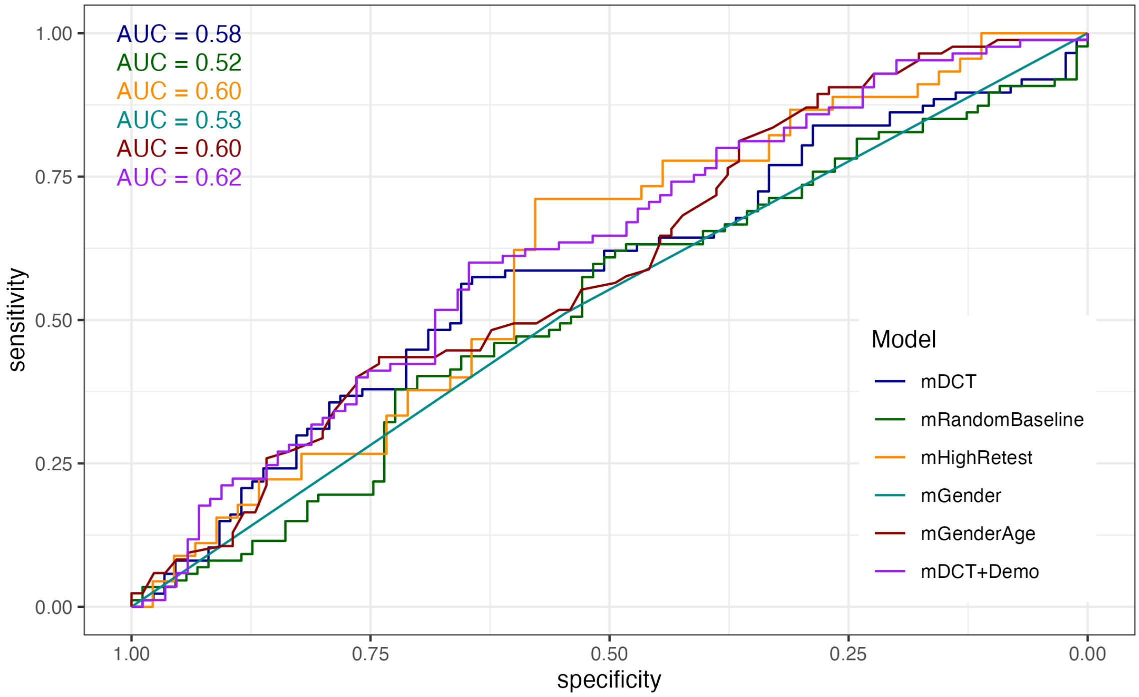

Figure 9: Study 2. Area Under the Curve (AUC) by model.

Figure S10: Study 2 - Confusion Matrices for All Models

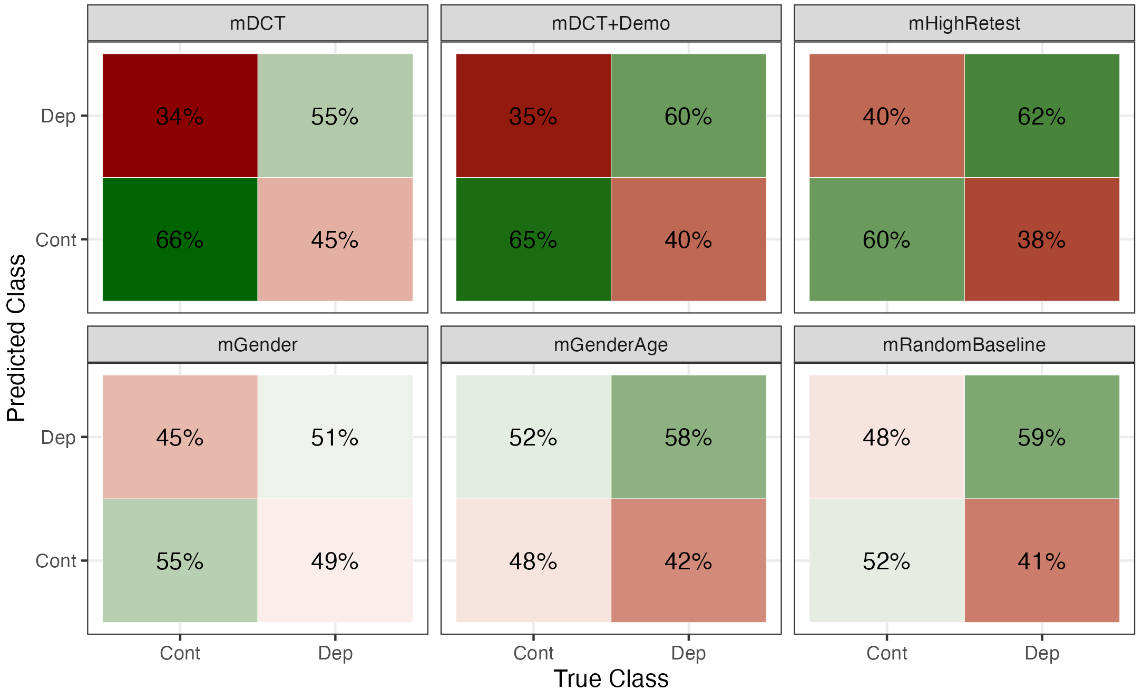

Figure 10: Study 2. Classification confusion matrices. Percentage correct predictions with respect to the true class. Red: lower values, green: higher values.

Figure S11: Correlation of Bootstrapped Item Classification Coefficients for *mDCT* in Study 1 and Study 2

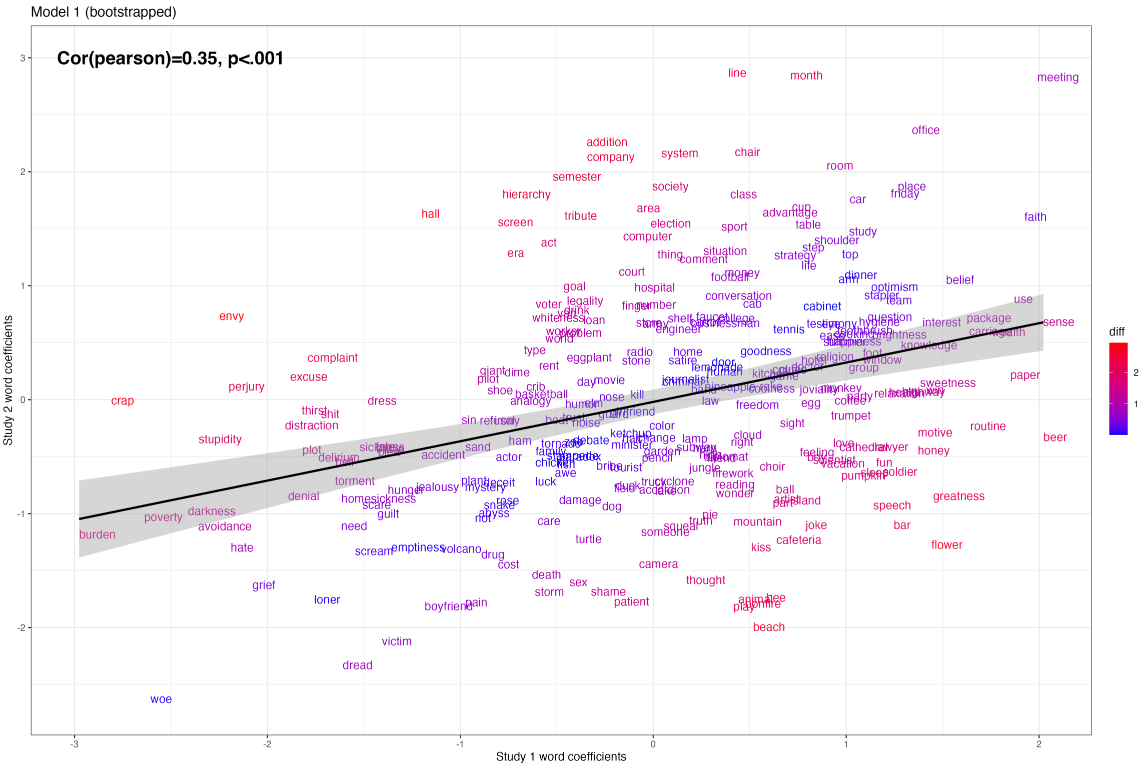

Figure 11: Correlation of average regression coefficients (all words) of mDCT in study 1 and study 2 (averaged across 1000 bootstraps). Words are colored according to the absolute difference between the effect in the two studies, where red indicates higher difference and blue indicates lower difference.

Figure S12: Correlation of Bootstrapped Item Classification Coefficients for *mDCT+Demo* in Study 1 and Study 2

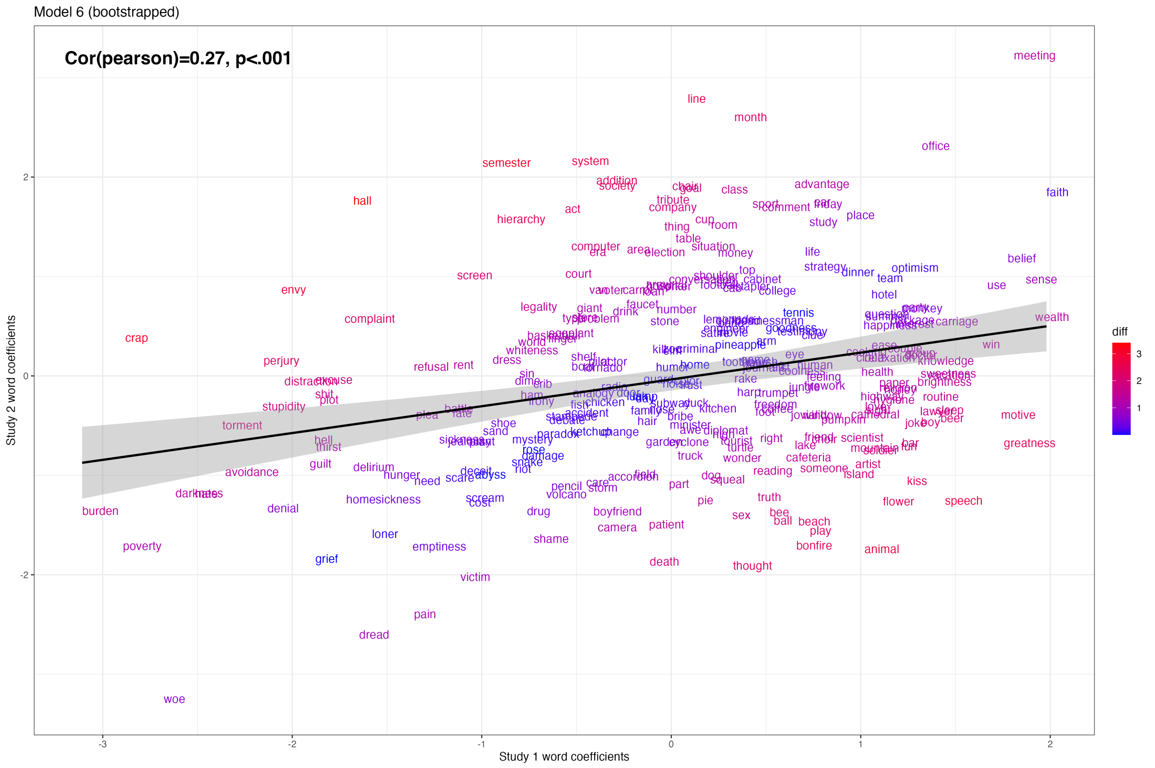

Figure 12: Correlation of average regression coefficients (all words) of mDCT+Demo in study 1 and study 2 (averaged across 1000 bootstraps). Words are colored according to the absolute difference between the effect in the two studies, where red indicates higher difference and blue indicates lower difference.

Figure S13: Correlation of Bootstrapped Item Classification Coefficients for *mRandomBaseline* in Study 1 and Study 2

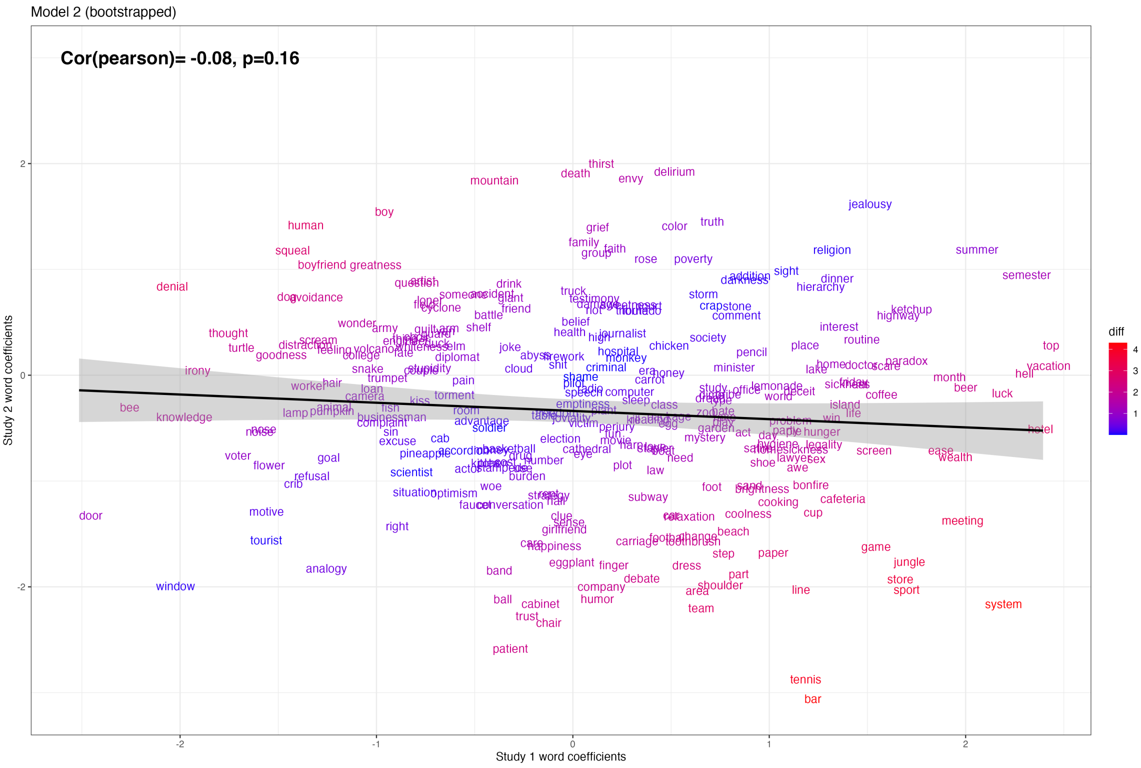

Figure 13: Correlation of average regression coefficients (all words) of mRandomBaseline in study 1 and study 2 (averaged across 1000 bootstraps). Words are colored according to the absolute difference between the effect in the two studies, where red indicates higher difference and blue indicates lower difference.

Table S5: Study 1 - PHQ-9 Sum Scores Predicted by Semantic Subject Profiles

Feature	Estimate	Error	95% CI.l	95% Ci.u
Valence	4,1	1,61	0,91	7,22
Arousal	1,18	2,05	-2,83	2,25
Dominance	3,17	1,88	-0,52	6,78
Anger	-4,45	1,34	-7,06	-1,87
Anticipation	2,82	1,49	-0,01	5,71
Disgust	-4,02	1,12	-6,21	-1,83
Fear	-3,98	1,19	-6,31	-1,64
Joy	5,66	1,42	2,86	8,42
Sadness	-3,46	0,93	-5,27	-1,64
Surprise	-1,55	1,38	-4,27	1,18
Trust	4,55	1,5	1,62	7,39

Table 5: Study 1. Results of Bayesian Regression Models predicting subject-level PHQ9 sum scores from semantic feature scores. Each model was estimated separately.

Table S6: Study 2 - PHQ-9 Sum Scores Predicted by Semantic Subject Profiles

Feature	Estimate	Error	95% CI.l	95% Ci.u
Valence	1,04	1,67	-2,24	4,32
Arousal	-1,6	2,16	-5,96	2,58
Dominance	0,37	2,07	-3,56	4,51
Anger	-3,79	1,29	-6,27	-1,24
Anticipation	-0,09	1,51	-3,04	2,88
Disgust	-3,01	1,08	-5,1	-0,91
Fear	-3,96	1,2	-6,29	-1,68
Joy	1,93	1,4	-0,79	4,72
Sadness	-3,22	0,91	-4,99	-1,41
Surprise	-2,05	1,3	-4,63	0,5
Trust	1,06	1,52	-1,92	4,08

Table 6: Study 2. Results of Bayesian Regression Models predicting subject-level PHQ9 sum scores from semantic feature scores. Each model was estimated separately.

1 Supplementary Experimental Procedures

1.1 Additions to preregistered protocol

The preregistered protocol along with all code for this study can be found at the online OSF repository (https://osf.io/bqhyg/). The submitted study adheres to the procedure described in the preregistrered protocol. However, few elements have been added to the analysis for further data scrunity. These include an additional model (mDCT+Demo) performing classification on PHQ-9 group from a combination of DCT responses, gender and age. This model was included as results suggested that while classification performance of demographic variables alone was poor, these variables do explain some variance in the PHQ-9. Hence, mDCT+Demo was added to assess whether the DCT-based classification model improved when accounting for gender- and age-related variance. Additionally, a baseline model (mRandomBaseline) was added, testing classification accuracy on a randomly shuffled version of the outcome variable (PHQ-9 class). This model served as benchmark for random classification against which the alternative models could be evaluated. Further, post-hoc parametric bootstrapping was applied to all models, as results revealed wide confidence intervals (CIs) for all classification models. Thus, bootstrapping was performed to assess the sensitivity of the models to specific random variation in the training data, and obtain a robust estimate of classification standard deviations. Lastly, two post-hoc analyses were performed, aiming to evaluate semantic effects of the relationship between DCT behavior and PHQ-9 scores. These included 1) the relationship between individual item (stimuli) classification coefficients and semantic features, and 2) the relationship between subject-wise semantic profiles and PHQ-9 scores. These allowed inferences on the extent to which general semantic features drive the DCT-based classification results, moving beyond effects at the individual word level.

1.2 Participants

The experiments were conducted on the online platform Prolific (`https://www.prolific.co`). All participants were native English speakers recruited through Prolific with minimum age of 18. No other inclusion criteria were defined. To ensure the quality of responses, subjects were excluded if they fulfilled at least one of three criteria indicating low effort responses: 1) reaction time (RT) below 300 ms. in more than 10% of the trials, 2) response (button) entropy below 0.80 indicating a consistent response pattern irrespective of the stimuli (figure 14, and and 3) more than 3 of 15 failed attention checks.

a. Entropy is calculated as following:

$$\mathrm{H(X)} = \sum_{k=0}^{n} \mathrm{P}(\mathrm{x}_i) log P(x_i)$$

Where *x* is the response variable with possibilities x_1 and x_2 (two button positions), and each response has probability:

$$P(x_i) = \frac{n(x_i)}{n}$$

where *n* is the total number of responses and *$n(x_i)$* is the number of responses *x_i*.

Figure 14: Entropy equation

Study 1 1004 subjects participated in study 1, of which 204 subjects were excluded due to missing data in either task- or questionnaire responses. Additionally, 8 subjects were excluded based on RT, 15 subjects were excluded based on response entropy, and 5 subjects were excluded based on attention check performance. Data exclusion yielded a final sample of 775 participants (gender: 352 female, 412 male, 10 non-binary, and 1 other; age: 159 were 18-29 years, 211 were 30-39 years, 147 were 40-49 years, 149 were 50-59 years, 107 were 60+ years, and 2 did not report age).

Study 2 1064 subjects participated in study 2, of which 155 subjects were excluded due to missing data in either task or questionnaire responses. Additionally, 5 subjects were excluded based on RT,

21 subjects were excluded based on response entropy, and 4 subjects were excluded based on attention check performance. Data exclusion yielded a final sample of 879 participants (gender: 410 female, 461 male, 6 non-binary, and 2 other; age: 213 were 18-29 years, 268 were 30-39 years, 190 were 40-49 years, 111 were 50-59 years, 92 were 60+ years, and 2 were unreported).

1.3 Materials

1.3.1 Demonstrative Choice Task (DCT)

Participants completed a 300-item Demonstrative Choice Task (DCT)[1]. For each trial an English noun was presented on the screen and participants were to match it with either a proximal ("this") or distal ("that") demonstrative forms by clicking one of two buttons presented below the stimulus. Participants were unaware of the purpose of the study. They were informed that there was no correct or incorrect answer, and were instructed to respond based on their immediate preference (Figure S**??** in Supplementary Materials). The task included 290 unique nouns, 10 of which were repeated twice, allowing measures of test-retest reliability. This yielded 300 trials in total. The 10 repeated nouns were selected at random for each participant. The order of stimulus presentation was randomized for each participant. The two response buttons were alternately oriented on a vertical or horizontal line, and response options ("this" or "that") switched positions at random. Thus, response options could take 2 x 2 (orientation x position) different spatial configurations. Following every 20th trial, participants responded to an attention check, in which they were to select the noun presented in trial t-1 from five options. The four incorrect options were chosen at random among the already presented stimuli. The task was conducted in three blocks of 100 trials, between which participants could take a short break. Average completion time was 18.96 min. and 18.80 min., for study 1 and 2, respectively. Choice of demonstrative form was coded as -1 (proximal) and 1 (distal).

DCT stimuli The current DCT was adapted from the original task presented in[1] to include nouns targeting depression- and personality-related differences. 290 unique nouns were included in total, of which 100 were targeting depression related differences, 50 were expected to be neutral, and 140 nouns were selected to target differences related to each of the BIG-5 personality traits.

Nouns targeting depression-related differences were selected based on analysis of data from a previous 480-item DCT study[1] including 2197 participants with associated PHQ-9 scores[2] of depression symptom severity. For each of the 480 words, the difference in proportion of proximal demonstrative choices between control subjects and individuals with depression (PHQ-9 sum ¿ 10) was computed, to identify and extract those yielding 1) the largest positive difference (subjects with depression were more likely to use proximal demonstrative than control subjects) (n=50), 2) the largest negative difference (control subjects were more likely to use proximal demonstrative than subjects with depression) (n=50), and 3) smallest absolute difference (neutral) (n=50). This generated 150 words included in the present DCT.

Selection of nouns targeting personality differences was based on an open-vocabulary analysis of 2467 stream-of-consciousness essays with associated Big 5 personality trait scores of the authors[3]. First, word-level features were extracted by computing the Anscombe-transformed normalized count occurrences[4] of each word in the full vocabulary, for each subject. Second, topic modeling was performed using Latent Dirichlet Analysis (LDA)[5] identifying 20 naturally occurring topics across all essays. The distribution of each topic in the essays were computed for every subject (following the method described in[6]). This resulted in a subject-wise feature vector of length 2484 (2464 word features; 20 topic features), representing the extent to which each word and topic was present in the essay. The features were inputted to a shallow neural network (NN) classifying the 5 personality traits (binary). The shallow NN allows inspection of the classification weights for the input features. Nouns for the current DCT were selected among the 30 most predictive positive and negative features (either words or topics) for each of the five personality traits. They were selected manually based on word class (nouns or convertible-to-nouns) and ensuring no duplicates. In total, 140 nouns were extracted yielding a final stimulus pool of 290 unique nouns.

1.3.2 Patient-Health Questionnaire 9-item (PHQ-9)

Depression symptom severity was measured with the 9-item Patient Health Questionnaire (PHQ-9)[2]. PHQ-9 is a self-administered version of the PRIME-MD diagnostic instrument and measures each of the 9 DSM-IV criteria for depression on a 4-point likert-scale ranging from 0 ("not at all") to 3 ("nearly every day"). The PHQ-9 instrument is routinely used to assess depression and has demonstrated robust validity and reliability[7,8], as well as sensitivity and specificity[9]. Standard

thresholds for mild, moderate, moderately severe, and severe depression are operationalized as a sum score of 5, 10, 15 and 20, respectively[2]. Previous validation analysis showed that a PHQ-9 score $\geq$ 10 yielded 88% sensitivity and 88% specificity for classifying cases of major depression diagnosed in mental health professional interviews[2]. A sum score $\geq$ 10 was defined as threshold for classification of participants into control- or depression group, coded as a factor with levels 0 and 1, respectively.

1.4 Analysis

All analyses were conducted in RStudio, version 4.1.1[10]. Study 1 and 2 were both analyzed according to the procedure described below.

1.4.1 Principal Component Analysis (PCA)

The 290 binary response features of the DCT were subjected to principal component analysis (PCA) to reduce dimensionality and correlation of the input features in subsequent classification models. PCA was conducted with the *stats* R package[10] which performs singular value decomposition and returns two matrices; an $m \ x \ d$ matrix with rotation weights of the m original variables on the d principal components (PCs), and an $n \ x \ d$ matrix with scores of the n subjects on the d PCs, calculated as the true data matrix multiplied by the rotation matrix. The subject-level PC scores were inputted as predictors in subsequent classification models.

In study 1, the fist 4 PCs captured most of the variance in the 290 DCT variables, with proportion of variance explained of 0.06 (SD=4.08), 0.03 (SD=2.89), 0.02 (SD=2.56) and 0.02 (SD=2.28), respectively. The remaining components each explained less than 1% of the total variance and had SDs below 1.6. The cumulative proportion of variance explained by the first 4 PCs was 14%, and the first 100 PCs explained 65% of the total variance.

In study 2, the first 4 PCs explained most of the variance in the 290 DCT variables, with proportion of variance explained of 0.05 (SD=3.66), 0.03 (SD=2.88), 0.02 (SD=2.57) and 0.02 (SD=2.16), respectively. The remaining components explained less than 1% of the total variance with SDs below 1.6. The cumulative proportion of variance explained by the first 4 PCs was 12%, and the first 100 PCs explained 63% of the total variance.

1.4.2 Classification analysis

Five logistic regression models were trained to predict depression group and evaluated and compared on out-of-sample classification performance. Prior to model estimation, the data was down-sampled by random seed to balance the prevalence of each outcome class, and subsequently partitioned into train- (=70%) and test sets (=30%) stratified by outcome group. This yielded a training set of n=342 and n=408 participants, for study 1 and 2, respectively, and a test set of n=144 and n=174 participants, for study 1 and 2, respectively. Model training was performed with the *caret* R package[11] using the *glm* method with k=3 repeated 10-fold cross-validation. Model performance was evaluated on out-of-sample classification accuracy, balanced between sensitivity (true positive rate), and specificity (true negative rate), and ROC AUC scores. Accuracy rate along with 95% confidence intervals for this rate were computed with a binomial test. P-values for classification performance were computed with a one-sided test, evaluating whether performance was better than the no information rate, taken to be the largest class percentage in the data. All above evaluation metrics were computed using the *caret* R package[11].

Model specifications Model 1 (mDCT) predicted depression class from principal component (PC) representation of DCT responses. 100 models were trained and evaluated, iteratively adding a PC as predictor, starting from 1 to the first 100 PCs. This allowed the model estimation procedure to identify the optimal number of PCs needed for the classification task. The model yielding best out-of-sample performance was identified and reported.

Classification effects of the individual DCT stimuli (nouns), e, were computed by matrix multi-

plication of the PCA rotation scores, w, and the model coefficients of the PCs, c:

$$\begin{bmatrix} e_1 \\ e_2 \\ \vdots \\ e_m \end{bmatrix} = \begin{bmatrix} w_{11} & w_{12} & \cdots & w_{1n} \\ w_{12} & w_{22} & \cdots & w_{2n} \\ \vdots & \vdots & \ddots & \vdots \\ w_{m1} & w_{m2} & \cdots & w_{mn} \end{bmatrix} \begin{bmatrix} c_1 & c_2 & \cdots & c_n \end{bmatrix} \tag{1}$$

where m is the individual DCT nouns (m=290) and n is the number of PCs included in the best model.

Model 2 (mHighRetest) addressed whether classification of depression from DCT responses improved when including only subjects exhibiting a test-retest reliability score above 70% on the 10 repeated stimuli (n=393 in study 1; n=434 in study 2). Test-retest reliability was defined as the percentage of the 10 repeated trials, for which the participant responded with the same demonstrative form. The model was trained and evaluated according to the procedure of mDCT. Classification effects of the individual DCT words were computed according to Eq. 1. After down-sampling and stratified data partitioning, the mHighRetest model was trained with n=162 and n=214 participants for study 1 and 2, respectively, and evaluated on n=68 and n=90 participants for study 1 and 2, respectively.

Model 3 (mDCT+Demo) modelled depression class as a function of DCT responses (*PCs*), *gender* and *age*. The mDCT+Demo model was performed only on subjects with reported gender of *male* or *female* and with no missing age data (yielding a sample of n=762 in study 1; n=867 in study 2). 100 models were trained and evaluated including the first 1 to 100 PCs as predictors in addition to *gender* and *age*. The model yielding best out-of-sample performance was identified and reported. Regression coefficients of individual DCT words were computed by Eq. 1. After down-sampling and stratified data-partitioning the mDCT+Demo model was trained on n=332 and n=402 subjects for study 1 and 2, respectively, and evaluated on n=140 and n=170 subjects for study 1 and 2 respectively.

Model 5 (mGenderAge) included both *gender* and *age* as predictors of depression group. Here, subjects for which age was not reported were additionally excluded from the analysis (yielding a sample size of n=762 in study 1, n=867 in study 2). After down-sampling and stratified data-partitioning mGender and mGenderAge were trained on n=332 and n=404, for study 1 and 2, respectively, and evaluated on n=140 and n=170 for study 1 and 2, respectively.

Lastly, to assess whether the DCT classifiers indeed learn non-random patterns related to depression symptom severity, their performance was compared to a random baseline model (mRandomBaseline). The random baseline model was defined identically to model 1, but trained on a randomly shuffled version of the outcome variable (depression class). The dependent variable thus represented random class labels across participants, while retaining the same distribution of group labels in the train- and test set.

1.4.3 Post-hoc Data Sensitivity Analysis

To assess robustness of model performance and word effects against random variability in the training data, a post-hoc sensitivity analysis was performed with bootstrapped model estimation. Each model was trained and evaluated on k=1000 new random partitions of the data into train and test sets (sampled with replacement). Model estimation and evaluation in each iteration followed the same procedure as described above for each model. However, as none of the best models included more than 50 PCs, the sensitivity analysis included only the first 1-50 PCs in model selection to reduce computational load. Mean accuracy score across the 1000 data partitions, along with SDs for the accuracy rate, were computed for each model.

1.4.4 Post-hoc Semantic Analysis of Word Effects

To address the extent to which word effects in the DCT classification models are associated with the semantic features of the words, Bayesian regression models (BRMs) were fitted predicting classification coefficients of the individual DCT items from their scores across the 11 semantic features of the NRC Emotion Lexicon[12] (*anger, anticipation, disgust, fear, joy, sadness, surprise* and *trust*) and the NRC-VAD lexicon[13] (*valence, arousal* and *dominance*). The analysis was performed on word coefficients from the mDCT+Demo model, which exhibited best performance in both studies. A

BRM was estimated for each semantic feature individually with 4 chains and 2000 iterations, using the *brms* R library. Results of word effects for the best model, mDCT+Demo, are reported in Figure S7. Results of bootstrapped word effects for mDCT+Demo are reported in Figure S8.

1.4.5 Post-hoc Semantic Subject Profiles Analysis

A post-hoc analysis was conducted to explore whether the semantic effects observed in the classification models can be captured in subject-wise semantic profiles based on DCT behavior. Bayesian regression models were fitted, evaluating the relationship between PHQ-9 sum scores and subject-wise semantic vectors. Each subject was ascribed a score on each of the 11 NRC-VAD semantic features, calculated by multiplying task responses (-1 or 1) for each word by the semantic feature score for each word, and taking the resulting mean for each feature. Each subject was thus represented by a semantic profile of 11 semantic feature scores. A linear BRM was fitted separately for each semantic feature as predictor of the continuous PHQ-9 sum score. Each BRM was estimated with 4 chains and 2000 iterations, using the *brms* R library.

References

[1] R. Rocca and M. Wallentin, "Demonstrative reference and semantic space: A large-scale demonstrative choice task study," *Frontiers in Psychology*, vol. 11, 2020, ISSN: 1664-1078. [Online]. Available: https://www.frontiersin.org/article/10.3389/fpsyg.2020.00629 (visited on 05/12/2022).

[2] K. Kroenke, R. L. Spitzer, and J. B. W. Williams, "The PHQ-9: Validity of a brief depression severity measure," *Journal of General Internal Medicine*, vol. 16, no. 9, pp. 606–613, 2001, _eprint: https://onlinelibrary.wiley.com/doi/pdf/10.1046/j.1525-1497.2001.016009606.x, ISSN: 1525-1497. DOI: 10.1046/j.1525-1497.2001.016009606.x. [Online]. Available: https://onlinelibrary.wiley.com/doi/abs/10.1046/j.1525-1497.2001.016009606.x (visited on 05/13/2022).

[3] J. W. Pennebaker and L. A. King, "Linguistic styles: Language use as an individual difference," *Journal of Personality and Social Psychology*, vol. 77, no. 6, pp. 1296–1312, 1999, Place: US Publisher: American Psychological Association, ISSN: 1939-1315. DOI: 10.1037/0022-3514.77.6.1296.

[4] H. A. Schwartz, J. C. Eichstaedt, M. L. Kern, L. Dziurzynski, S. M. Ramones, M. Agrawal, A. Shah, M. Kosinski, D. Stillwell, M. E. P. Seligman, and L. H. Ungar, "Personality, gender, and age in the language of social media: The open-vocabulary approach," *PLoS ONE*, vol. 8, no. 9, T. Preis, Ed., e73791, Sep. 25, 2013, ISSN: 1932-6203. DOI: 10.1371/journal.pone.0073791. [Online]. Available: https://dx.plos.org/10.1371/journal.pone.0073791 (visited on 05/13/2022).

[5] D. M. Blei, A. Y. Ng, and M. I. Jordan, "Latent dirichlet allocation," *Journal of Machine Learning Research 3*, vol. 3, pp. 993–1022, 2003.

[6] G. Park, H. A. Schwartz, J. C. Eichstaedt, M. L. Kern, M. Kosinski, D. J. Stillwell, L. H. Ungar, and M. E. P. Seligman, "Automatic personality assessment through social media language.," *Journal of Personality and Social Psychology*, vol. 108, no. 6, pp. 934–952, Jun. 2015, ISSN: 1939-1315, 0022-3514. DOI: 10.1037/pspp0000020. [Online]. Available: http://doi.apa.org/getdoi.cfm?doi=10.1037/pspp0000020 (visited on 05/13/2022).

[7] I. M. Cameron, J. R. Crawford, K. Lawton, and I. C. Reid, "Psychometric comparison of PHQ-9 and HADS for measuring depression severity in primary care," *British Journal of General Practice*, vol. 58, no. 546, pp. 32–36, Jan. 1, 2008, Publisher: British Journal of General Practice Section: Original Papers, ISSN: 0960-1643, 1478-5242. DOI: 10.3399/bjgp08X263794. [Online]. Available: https://bjgp.org/content/58/546/32 (visited on 08/31/2022).

[8] S. Maroufizadeh, R. Omani-Samani, A. Almasi-Hashiani, P. Amini, and M. Sepidarkish, "The reliability and validity of the patient health questionnaire-9 (PHQ-9) and PHQ-2 in patients with infertility," *Reproductive Health*, vol. 16, no. 1, p. 137, Dec. 2019, ISSN: 1742-4755. DOI: 10.1186/s12978-019-0802-x. [Online]. Available: https://reproductive-health-journal.biomedcentral.com/articles/10.1186/s12978-019-0802-x (visited on 08/31/2022).

[9] B. Levis, A. Benedetti, and B. D. Thombs, "Accuracy of patient health questionnaire-9 (PHQ-9) for screening to detect major depression: Individual participant data meta-analysis," *BMJ*, vol. 365, p. l1476, Apr. 9, 2019, Publisher: British Medical Journal Publishing Group Section: Research, ISSN: 0959-8138, 1756-1833. DOI: 10.1136/bmj.l1476. [Online]. Available: https://www.bmj.com/content/365/bmj.l1476 (visited on 08/31/2022).

[10] R. Team, *RStudio: Integrated development for r.* RStudio, 2020. [Online]. Available: http://www.rstudio.com/.

[11] M. Kuhn, "Building predictive models in r using the caret package," *Journal of Statistical Software*, vol. 28, pp. 1–26, Nov. 10, 2008, ISSN: 1548-7660. DOI: 10.18637/jss.v028.i05. [Online]. Available: https://doi.org/10.18637/jss.v028.i05 (visited on 05/13/2022).

[12] S. M. Mohammad, *Word affect intensities*, Oct. 15, 2022. DOI: 10.48550/arXiv.1704.08798. arXiv: 1704.08798[cs]. [Online]. Available: http://arxiv.org/abs/1704.08798 (visited on 11/29/2022).

[13] S. Mohammad, "Obtaining reliable human ratings of valence, arousal, and dominance for 20,000 english words," in *Proceedings of the 56th Annual Meeting of the Association for Computational Linguistics (Volume 1: Long Papers)*, Melbourne, Australia: Association for Computational Linguistics, Jul. 2018, pp. 174–184. DOI: 10.18653/v1/P18-1017. [Online]. Available: https://aclanthology.org/P18-1017 (visited on 11/29/2022).
